# Digital Monitoring and Chronic Pain Trajectories in Patients Receiving Tertiary Pain Care

**DOI:** 10.1001/jamanetworkopen.2026.27217

**Published:** 2026-08-04

**Authors:** Nicolas Kerckhove, Armel Soubeiga, Jules Phalip, Célian Bertin, Bastien Doreau, Xavier Moisset, Noémie Delage, Nicolas Authier, Violaine Antoine, Bruno Pereira

**Affiliations:** 1 Université Clermont Auvergne, INSERM, UMR 1107, NEURO-DOL, CHU Clermont-Ferrand, Service de Pharmacologie Médicale, Service de médecine de la douleur, Service de Neurologie, Direction de la Recherche Clinique et de l’Innovation, Clermont-Ferrand, France; 2 Fondation Analgesia, Clermont-Ferrand, France; 3 Université Clermont Auvergne, CNRS, Mines Saint-Etienne, Clermont-Auvergne-INP, LIMOS, Clermont-Ferrand, France

## Abstract

**Importance:**

Chronic pain management relies on baseline prognostic models, although digital monitoring may better capture long-term patient trajectories.

**Objective:**

To describe 6-month chronic pain trajectories using weekly assessments and to evaluate the value of routinely collected baseline sociodemographic and clinical variables in estimating trajectories.

**Design, Setting, and Participants:**

This multicenter mobile health cohort study was conducted in French tertiary pain clinics between September 2019 and 2025. Adults with chronic noncancer pain initiating follow-up in a pain clinic and completing at least 6 months of follow-up were eligible.

**Exposure:**

Participation in the eDOL digital health program with weekly assessments alongside routine care.

**Main Outcomes and Measures:**

The primary outcomes were weekly bodily comfort, sleep, and mood measured on 11-point numeric rating scale and aggregated into monthly medians. Trajectory stability, time spent in predefined clinical states, latent trajectory classes, and performance of baseline variables in estimating outcomes were assessed using linear mixed-effects models, time-in-state analyses, gaussian mixture models , and machine learning. Informative attrition was addressed using inverse probability of censoring weighting and joint longitudinal-survival modeling.

**Results:**

Among 1477 participants (76.8% [95% CI, 74.6%-78.9%] aged 35-64 years; 82.1% [95% CI, 77.9%-86.3%] women; median pain duration >5 years), most were receiving multimodal treatment for multimorbid chronic pain. Average trajectories remained stable, with no clinically meaningful change in patient-reported outcomes. Three trajectory classes were identified: stable (1266 participants; 85.7% [95% CI, 83.9%-87.5%]), improving (61 participants; 4.1% [95% CI, 3.2%-5.2%]), and worsening (150 participants; 10.2% [95% CI, 8.6%-11.7%]). Baseline characteristics had limited value in estimating pain trajectories.

**Conclusions and Relevance:**

In tertiary pain clinics patients, trajectories of bodily comfort, sleep, and mood were predominantly stable over 6 months. Baseline characteristics poorly estimated long-term trajectories, supporting continuous digital monitoring and individualized change detection rather than baseline-only prognostic stratification.

## Key Points

**Question:**

Can high-frequency digital monitoring reveal clinically meaningful chronic pain trajectory patterns, and can routinely available baseline variables be used to estimate long-term evolution?

**Findings:**

In this cohort study of 1477 patients with chronic noncancer pain observed weekly for 6 months, trajectories of bodily comfort, sleep, and mood were predominantly stable; only 4.1% of patients improved and 10.2% worsened. Baseline variables provided no clinically meaningful estimate of trajectory membership.

**Meaning:**

These findings suggest that chronic pain in tertiary care is better conceptualized as a stable digital state with rare transitions, supporting continuous monitoring and individualized deviation detection over baseline-only prognostic stratification.

## Teaser

This cohort study describes 6-month chronic pain trajectories using weekly assessments and evaluates the value of routinely collected baseline sociodemographic and clinical variables in estimating trajectories.

## Introduction

Chronic pain affects approximately 1 in 5 adults worldwide, with substantial health care costs attributable to its persistence, functional impairment, and biopsychosocial complexity.^1,2^ Despite advances in understanding nociceptive, nociplastic, neuropathic, and central sensitization mechanisms, estimating long-term clinical evolution remains challenging. Mobile health applications offer a unique opportunity to capture high-resolution longitudinal data on symptoms, functional impact, and psychological states in real time,^3,4^ enabling detection of subtle intraindividual fluctuations that traditional clinic-based assessments cannot detect.^5,6^

Previous longitudinal cohort studies^7,8,9,10^ using infrequent assessments have been limited by small sample sizes, single-pain focus, and inadequate handling of missing data and attrition bias.^11,12^ Attrition is particularly problematic in mobile health studies, where engagement is voluntary and often declines rapidly.^13,14^ Recent advances in trajectory modeling, such as transformer-based approaches,^15^ rely on population-scale cohorts with dense multimodal histories, conditions not typically available in general population mobile health or routine pain-clinic settings. Thus, identifying distinct trajectory subgroups and assessing the value of routinely available baseline clinical data in estimating trajectories are further challenges in chronic pain management that this study aimed to address.

## Methods

### Study Design and Participants

This study is reported in accordance with the Strengthening the Reporting of Observational Studies in Epidemiology (STROBE) reporting guidelines for cohort studies. It is a secondary analysis of the eDOL mobile health cohort,^14^ a web platform and mobile application for the long-term follow-up of patients with chronic noncancer pain, conducted in French tertiary pain clinics from September 2019 to September 2025. All participants provided written informed consent; the study was approved by the relevant research ethics committee and the French Data Protection Authority and was registered at ClinicalTrials.gov (NCT04880096), in accordance with the Declaration of Helsinki.^16^ Eligible participants had at least 6 months of follow-up and baseline data available within the first month (full details are shown in the eMethods in Supplement 1).

### Data Collection and Outcomes

Three patient-reported outcome measures (PROMs)—bodily comfort, sleep, and mood—were selected to capture the multidimensional biopsychosocial experience of chronic pain over 6 months. Bodily comfort is a weekly numeric rating scale item designed to capture the patient’s overall physical well-being, including but not limited to pain. To simplify its use by patients, its implementation in the eDOL tool, and its analysis, it was conceptually derived from the Patient Reported-Outcomes Measurement Information System (PROMIS) Physical Function, which assesses how pain affects physical functioning and general sense of bodily well-being. Similarly, the sleep item was inspired by the PROMIS sleep disturbance, and the mood item draws from the PROMIS depression. These items were selected as weekly ecological momentary assessments within the eDOL smartphone application, intended to provide a parsimonious, biopsychosocial monitoring framework compatible with high-frequency digital collection. Each item was reported weekly on an 11-point numeric rating scale (0 = poor; 10 = high quality).

Pain intensity was intentionally excluded as a primary outcome: it is increasingly recognized as an incomplete proxy for the chronic pain experience and is often poorly correlated with functional disability and quality of life in tertiary care settings.^17,18,19,20^ The biopsychosocial combination of bodily comfort, sleep, and mood aligns with Initiative on Methods, Measurement, and Pain Assessment in Clinical Trials recommendations^20^ and better reflects the domains patients themselves prioritize.

To mitigate measurement noise and short-term variation, data were aggregated to monthly medians (up to 6 time points), following established recommendations for high-frequency ecological momentary assessment data.^21^ This approach reduces the impact of sporadic missing observations and stabilizes trajectory estimation without eliminating clinically meaningful changes.

### Study Objectives

The primary objectives were to (1) characterize the stability and variability of patient-reported outcomes across bodily comfort, sleep, and mood over a 6-month period; (2) identify latent trajectory classes using advanced clustering techniques; and (3) evaluate the capacity of baseline sociodemographic and clinical variables to estimate trajectories using machine learning, accounting rigorously for missing data and informative attrition.^22,23^ Additional details are shown in eFigure 1 in Supplement 1.

### Statistical Analysis

All analyses were performed using R statistical software version 4.4 (R Project for Statistical Computing). The underlying R code for this study has been deposited in a public repository.^24^ Descriptive statistics are presented as means (SDs) for continuous variables and counts with proportions (95% CI) for categorical variables. A 2-sided significance level of α = .05 was applied throughout.

Longitudinal trajectories were estimated using linear mixed-effects models (LMMs) with random intercepts and slopes for each patient. Informative attrition was addressed using 2 complementary approaches: inverse probability of censoring weighting (IPCW), a logistic regression correcting for systematic differences between dropouts and completers based on baseline covariates (age, sex, pain duration, and PROMIS interference score),^23,25^ and joint longitudinal-survival models linking LMM score trajectories with a Cox submodel for dropout risk.^12,22^ The 4-week terminal window (weeks 24-28) defined nonadherence, consistent with prior eDOL cohort studies.^14^ Missing data were handled using multiple imputation by chained equations with estimating mean matching (50 imputed datasets and 10 iterations).^26,27,28,29^ Intraindividual dynamics were assessed using a time-in-state approach applied to raw weekly nonaggregated data, classifying each weekly observation as improvement, stability, or decline^30^ on the basis of score changes of at least plus or minus 1.5 points, a conservative threshold derived from minimal clinically important difference estimates in chronic musculoskeletal and nociplastic pain.^31,32^

Latent trajectory classes were identified using multivariable gaussian mixture models (GMMs), a probabilistic clustering approach grouping patients with similar 6-month outcome profiles, allowing flexible cluster shapes and soft probabilistic class assignments that reflect the inherent uncertainty in behavioral health states. Prior to clustering, subject-specific random effects (best linear unbiased predictors [BLUPs] extracted from LMMs) were reduced using principal component analysis (PCA) to address dimensionality and multicollinearity. We also evaluated k-means clustering applied to baseline PROMs and PCA-transformed BLUPs as a complementary approach. Models with 1 to 4 classes were compared using the bayesian information criterion, entropy, and odds of correct classification (threshold >5). Monte Carlo simulations (n = 2000) generated 95% CIs for estimated trajectories. Class clinical profiles were characterized using the categories description procedure within a hierarchical clustering on principal components framework, which identifies variables significantly discriminating each cluster from the overall population via V tests (significant at V > 1.96).^33^

Estimating models for trajectory class membership were developed using K-nearest neighbors, support vector machines, random forest, gradient boosting, categorical boosting, and multinomial logistic regression, with hyperparameter optimization via grid search and performance assessed through 10-fold cross-validation. Metrics robust to class imbalance were prioritized, including Cohen κ, balanced accuracy, and the area under the precision-recall curve (full details are shown in the eMethods in Supplement 1).

## Results

### Study Population

**Figure 1.**
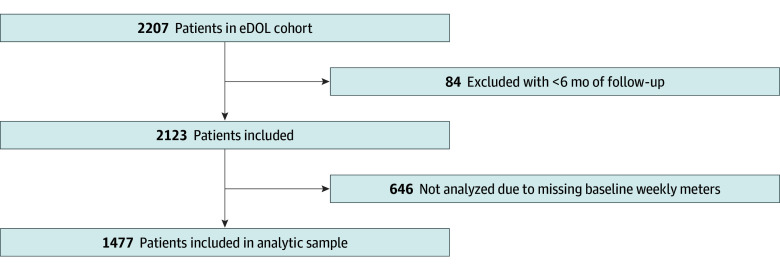
Flowchart of Participant Enrollment Figure shows participant selection from the initial eDOL cohort (n = 2207) to the analytical sample included in trajectory analyses (n = 1477), including exclusion criteria and availability of longitudinal patient-reported outcome measures data.

Among 2207 initial eDOL users, 1477 met the inclusion criteria (Figure 1). Most participants were middle-aged (76.8% [95% CI, 74.6%-78.9%] aged 35-64 years), women (82.1% [95% CI, 77.9%-86.3%]), overweight, and professionally active, with one-fifth on sick leave due to pain. Long-standing pain exceeding 5 years was reported in the majority of cases, and nociplastic (mainly fibromyalgia) and musculoskeletal pain were the most prevalent diagnoses. The main analgesic pharmacological treatments were antidepressants, paracetamol, and antiepileptic drugs, and the main nonpharmacological treatments were neuromodulation and physical or manual therapies. High psychological comorbidity was observed, with participants reporting psychiatric disorders (including anxiety and depression) and substance use disorders (including nicotine dependence), while a large majority had a history of other medical conditions. Comparisons between the analytical sample and the initial cohort revealed no significant differences in baseline characteristics (eTable 1 in Supplement 1).

### Attrition and Missing Data Bias

Attrition was high from the first weeks of follow-up and stabilized after 2 months (eFigure 2 in Supplement 1). At month 6, 602 of 1477 patients (40.8%; 95% CI, 38.3%-43.3%) had completed PROMs and were considered as adherent patients. Over the entire follow-up period, 73.6% (95% CI, 71.3%-75.8%) of patients had more than 50% of weekly data missing, a proportion that decreased to 59.9% (95% CI, 57.4%-62.4%) after monthly aggregation. Comparison of baseline characteristics between adherent and nonadherent patients did not identify variables significantly associated with dropout (eTable 2 in Supplement 1). However, the observed association between clinical trajectory and dropout motivated the use of IPCW and joint models to rigorously account for informative attrition and minimize potential selection bias.

### Overall Trajectories

LMMs and joint models adjusted for informative attrition consistently showed stable average trajectories across all 3 PROMs. Bodily comfort changed by approximately −0.01 point per month after IPCW correction, sleep changed by −0.03 point per month, and mood increased slightly by 0.02 point per month, with none reaching statistical significance. Results were consistent across uncorrected, IPCW, and joint model approaches (eFigure 3 in Supplement 1).

### Dynamic Classification Using Time-in-State

**Figure 2.**
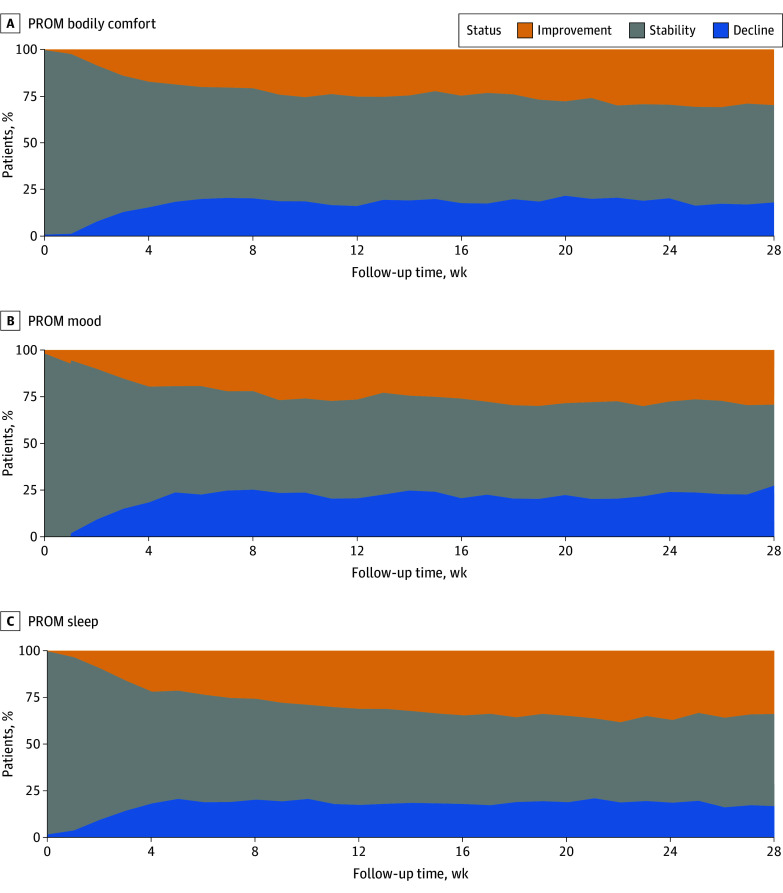
Area Graph of Trajectory Dynamics Before Study Disengagement Graph shows weekly distribution of changes in patient-reported outcome measure (PROM) scores between the first and last available observation prior to dropout, using a plus or minus 1.5 point threshold to define improvement, decline, or stability for patients with less than 50% missing data and no imputation (see eFigure 4 in Supplement 1 for other scenarios).

Time-in-state analysis, conducted on raw weekly nonaggregated data, confirmed stability as the predominant state across all 3 PROMs, all missing data thresholds, and all imputation strategies. For patients with less than 50% missing data and without imputation, on average across PROMs and the 28 weeks of follow-up, a majority (mean [SD], 57.1% [13.0%]) who discontinued follow-up displayed consistent stability preceding dropout (Figure 2). Similar results were observed for others scenarios (eFigure 4 in Supplement 1).

### Longitudinal Clustering of Trajectories

We found that k-means clustering based on baseline features primarily separated individuals by their initial PROM levels without capturing longitudinal patterns (eFigure 5A in Supplement 1); applied to PCA-transformed BLUPs, it failed to yield clinically meaningful clusters (eFigure 5B in Supplement 1). Importantly, k-means applied directly to raw weekly nonaggregated data across 5 missing data thresholds confirmed stable, flat trajectories within all clusters for all 3 PROMs, regardless of imputation strategy (eFigure 6 in Supplement 1). GMM applied to monthly PROM summaries provided more distinct and interpretable trajectory groups and was retained as the primary method.

**Figure 3.**
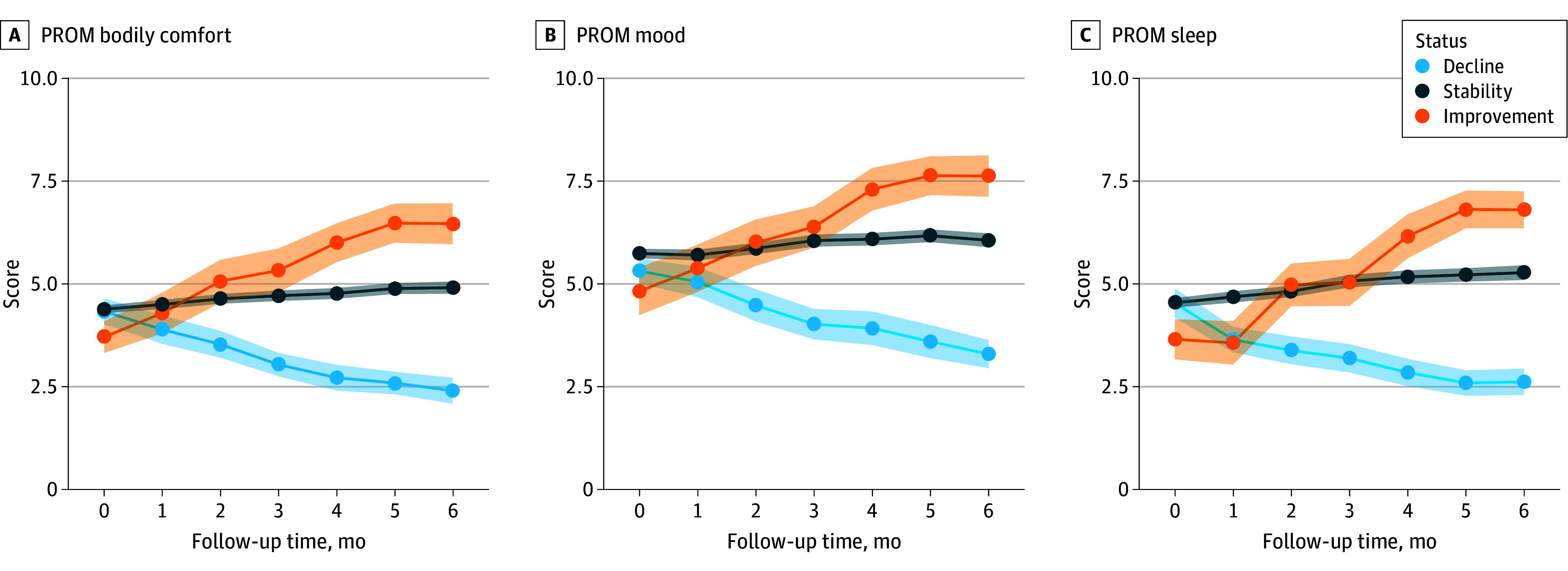
Line Graphs of Latent Trajectory Classes Identified Using Gaussian Mixture Models Observed patient-reported outcome measure (PROM) values and model-estimated trajectories for the 3 latent classes identified by the multivariable GMM analysis: stable, improvement, and decline trajectories. Lines represent model-estimated mean trajectories for each class. Shaded bands represent 95% CIs around the observed class means at each time point.

The bayesian information criterion decreased substantially from 1 to 3 classes (73 681.55 to 73 327.30); a fourth class yielded negligible improvement (bayesian information criterion, 73 326.53) and created a microcluster comprising 0.6% of patients, indicating model overfitting (eTable 3 in Supplement 1). The 3-class model was, therefore, selected. The majority followed a stable trajectory (class 1, 1266 patients; 85.7%; 95% CI, 83.9%-87.5%). Two distinct minority phenotypes were identified: class 2 (61 patients; 4.1%; 95% CI, 3.2%-5.2%), showed rapid clinical improvement (slope mean [SD], 2.25 [0.04] points per 6 months; *P* < .001 vs baseline and class 1), and class 3 (150 patients; 10.2%; 95% CI, 8.6%-11.7%), showed gradual decline (slope mean [SD], −1.42 [0.03] points per 6 months; *P* < .001 vs baseline and class 1) (Figure 3 and Table 1). Despite class imbalance, the model demonstrated high discriminatory power for minority clusters, with mean posterior probabilities exceeding 0.70 and odds of correct classification values well above the threshold of 5.0 (68.4 and 27.7 for classes 2 and 3, respectively).

**Table 1.** Estimated Trajectory Slopes for PROMS According to GMM Classes^a^

GMM class	No. of patients (%) [95% CI]	PROMs evolutions, mean (SE) [95% CI], slope/6 mo	*P* value vs baseline	*P* value vs class 1
1	1266 (85.7) [83.9 to 87.5]	0.34 (0.01) [0.32 to 0.36]	<.001	NA
2	61 (4.1) [3.1 to 5.1]	2.25 (0.04) [2.17 to 2.33]	<.001	<.001
3	150 (10.2) [8.6 to 11.7]	−1.42 (0.03) [−1.48 to −1.36]	<.001	<.001

Abbreviations: GMM, Gaussian mixture model; NA, not applicable; PROM, patient-reported outcome measure.

^a^ Data are monthly slopes of bodily comfort, sleep, and mood scores estimated for each latent trajectory class identified by the GMM. Positive slopes indicate improvement, whereas negative slopes indicate decline.

Class 2 (improvement) patients had slightly more degraded PROMs at inclusion (V = −3.0 to −2.8), but significantly lower anxiety (V = −2.6) and depression (V = −2.4) scores, and a higher prevalence of pain duration exceeding 10 years (V = 2.3) and divorce (V = 2.5). Class 3 (decline) was characterized by the most degraded multidimensional profiles, including sleep, cognition, catastrophizing, anxiety, depression, and kinesophobia (V ranging from 3.3 to 6.3), and higher social precariousness (V = 3.3), greater disability allowance receipt (V = 2.7), and reduced quality of life (V = −3.1). Both minority classes were characterized by substantially higher adherence rates (V = 5.3 and 4.1). See Table 2 for descriptive results.

**Table 2.** Clinical and Sociodemographic Characteristics of Minority Trajectory Classes^a^

Significant variables related to classes^b^	GMM class 2 (n = 61)			Class 3 (n = 150)			All patients (N = 1477)
	Score, mean (SD)	V test^c^	Effect size, Cohen *d* or Cohen *h*	Score, mean (SD)	V test	Effect size, Cohen *d* or Cohen *h*	
Adherent patients, % (95% CI)	73.8 (62.7 to 84.8)	5.29^d^	0.68^d^	56.6 (48.7 to 64.6)	4.14^d^	0.32^d^	40.8 (38.3 to 43.3)
Alexythymia (100)	61.3 (9.3)	−0.59	−0.07	64.2 (11.3)	2.64^d^	0.20^d^	62.1 (10.6)
Anxiety (21)	8.7 (3.7)	−2.63^d^	−0.33^d^	11.1 (4.3)	3.35^d^	0.26^d^	10.0 (4.0)
Patient-reported outcome measures							
Bodily comfort (10)	3.7 (1.6)	−2.89^d^	−0.36^d^	4.2 (2.0)	−0.76	−0.06	4.3 (1.7)
Mood (10)	4.9 (2.2)	−2.76^d^	−0.35^d^	5.2 (2.0)	−2.53^d^	−0.19	5.6 (2.1)
Sleep (10)	3.7 (1.9)	−3.04^d^	−0.38^d^	4.5 (2.3)	−0.16	−0.01	4.5 (2.1)
Catastrophizing (52)	26.3 (10.5)	0.00	0.00	30.3 (11.5)	4.63^d^	0.36^d^	26.3 (11.0)
Cognition (10)	4.6 (3.0)	−0.98	−0.12	6.2 (2.4)	5.89^d^	0.45^d^	4.9 (2.7)
Depression (21)	6.8 (3.7)	−2.42^d^	−0.30^d^	9.4 (4.0)	4.43^d^	0.34^d^	8.0 (4.1)
Health-related quality of life (100)	46.0 (21.1)	−1.00	−0.13	43.8 (19.6)	−3.12^d^	−0.24^d^	48.6 (19.9)
Injustice (48)	27.4 (9.8)	0.15	0.02	30.7 (10.2)	4.51^d^	0.35^d^	27.2 (10.1)
Kinesophobia (68)	43.8 (5.9)	1.76	0.22^d^	45.0 (8.6)	4.77^d^	0.37^d^	41.9 (8.3)
Life event: divorce, % (95% CI)	19.7 (9.7 to 29.6)	2.50^d^	0.30^d^	10.7 (5.7 to 15.6)	0.51	0.04	9.4 (7.9 to 10.9)
Disability allowance, % (95% CI)	4.9 (−0.5 to 10.3)	−1.21^d^	−0.17^d^	16.0 (10.1 to 21.9)	2.68	0.21	9.3 (7.8 to 10.8)
Pain duration ≥10 y, % (95% CI)	50.8 (38.3 to 63.4)	2.26^d^	0.28^d^	34.0 (26.4 to 41.6)	−0.88	−0.07	36.8 (34.3 to 39.2)
Pain intensity (10)	3.9 (1.2)	−1.02	−0.13	4.6 (1.3)	5.28^d^	0.41^d^	4.0 (1.3)
Pain interference (10)	5.8 (1.8)	0.07	0.01	6.4 (1.8)	3.79^d^	0.29^d^	5.8 (1.9)
Sleep disorders (100)	54.5 (19.1)	−0.54	−0.07	63.9 (16.1)	6.28^d^	0.48^d^	55.6 (17.0)
Social vulnerability (100)	33.4 (24.0)	1.02	0.13	36.0 (22.3)	3.26^d^	0.25^d^	30.8 (20.2)

^a^ Class 1 (85.7% of patients) was not presented because these characteristics are similar to the overall population (no effect size >0.2).

^b^ Numbers in parentheses denote maximum scores.

^c^ Compared with overall patients. Variables significantly associated with the improvement and/or decline classes identified by the categories description procedure. V test statistics indicate the degree to which each variable is overrepresented or underrepresented in the cluster compared with the overall cohort. Only variables with an effect size greater than 0.2 or less than −0.2 and significant V test results are shown.

^d^ Denotes statistical significance.

### Trajectory Estimates

Standard algorithms (random forest, support vector machines, k-nearest neighbors, gradient boosting, and multinomial logistic regression) demonstrated majority class bias, with high overall accuracy (>85%) but near-chance balanced accuracy (approximately 0.50) and κ value (0.00 to 0.05). Categorical boosting with cost-sensitive class weighting (1:5:3) achieved the best performance (balanced accuracy, 0.58; sensitivity, 0.44; κ, 0.16). Performance remained similar across the synthetic minority oversampling technique, balanced random forest, and extensive hyperparameter tuning (eTable 4 in Supplement 1).

## Discussion

This large digital cohort study establishes 3 fundamental findings about chronic pain trajectories in tertiary care. First, weekly monitoring reveals predominant clinical stability, with patients maintaining stable biopsychosocial profiles over 6 months. Second, meaningful transitions occurred in a minority and were concentrated in the first months of specialized care initiation. Third, baseline sociodemographic and clinical variables provided no clinically meaningful estimate of these transitions, even with advanced machine learning and attrition correction.^1,34^ This predominance of stability should not be generalized beyond tertiary care or short-term follow-up, as longer-term studies have reported varied trajectories.^8,10,35^

The observed stability aligns with prior literature suggesting that pain intensity and functional impact fluctuate less than theoretical models imply.^7,8,9,10,35^ Unlike studies relying on sparse measurements, the high temporal resolution of the eDOL cohort supports stability as an intrinsic feature, rather than an artifact, of infrequent follow-up.^3,21^ Crucially, stability should not be interpreted as absence of change, but as a dynamic equilibrium around an individual baseline, consistent with the concept of a dynamic attractor in chronic pain.^36^ These observations carry direct health economics implications. In tertiary care, where patients are referred precisely because prior treatments have failed, preventing further deterioration over 6 months represents a meaningful therapeutic achievement. The 10.2% experiencing worsening pain, identifiable at baseline by elevated psychological burden and social precariousness, represent the priority target for intensified intervention.

Compared with recent mobile health studies, our findings highlight an even higher prevalence of stable trajectories. Although Little et al^7^ identified that a majority of patients (66%) followed a stable pain trajectory, our multivariable analysis revealed a substantially higher proportion of stability. This higher rate of stability in our cohort confirms that, in the context of tertiary care (vs primary care in the study by Little et al^7^) and long-term monitoring, the vast majority of users maintain consistent symptom profiles over a 6-month period. This high stability rate provides a robust baseline for identifying the minority of patients experiencing clinically meaningful change. The presence of smaller groups showing improvement or worsening is also consistent with prior longitudinal findings in musculoskeletal low-back pain and neuropathic pain.^8,9,10^ The use of multivariable GMM was critical in capturing a more holistic clinical phenotype by integrating multiple PROMs simultaneously. Despite class imbalance, the robustness of minority clusters was supported by high odds of correct classification values.^37^ Moderate entropy reflects the clinical reality of chronic pain, where transitions are gradual and not sharply delineated. These classes should therefore be interpreted as trajectory archetypes rather than strict patient categories.

The inability of baseline variables to estimate trajectory membership, despite large sample size, advanced algorithms, attrition correction, and class imbalance handling, is a clinically meaningful negative finding. Apparent accuracy was driven by class imbalance; balanced accuracy and κ values remained low, a known pitfall in imbalanced clinical datasets.^38^ These results are consistent with evidence that even state-of-the-art transformer-based approaches (eg, Delphi-2M, trained on >2 million patients),^15^ require massive multimodal longitudinal histories unavailable in routine clinical settings: estimating gains derive from data richness, not algorithm complexity alone.

These findings reframe mobile health utility in chronic pain management. Rather than baseline prognostic stratification, which was demonstrably ineffective here, this suggests that trajectory changes are likely driven by dynamic, unmeasured factors occurring during follow-up, such as treatment changes, comorbidities, or life events.^1,39^ Weekly monitoring creates digital phenotypes amenable to anomaly detection, with personalized thresholds flagging deviations from established stability patterns. The early concentration of transitions (first 2-3 months) suggests optimal alert timing, while stability-dropout linkage identifies engagement risk. Immediate clinical applications follow directly. Stability constitutes therapeutic success in tertiary care, where baseline severity is high and complete resolution rare. Monitoring dashboards could prioritize the minority experiencing change. Such just-in-time adaptive interventions^40^ could deliver automated peer support to improvers, urgent specialist triage for decliners, and engagement prompts during stability. Combining active PROMs with passive sensing (accelerometry or sleep patterns) would enhance signal precision without patient burden. In this context, mobile health platforms may be better suited as tools for continuous monitoring rather than prediction.^3^

### Strengths and Limitations

A major strength lies in the convergent evidence from complementary methods (LMM, IPCW, joint model, time-in-state, and GMM on monthly data), ensuring robustness despite high attrition. The inception cohort design, enrolling only patients at the start of specialized pain care, reduces heterogeneity from prior treatment exposure and provides a cleaner view of early tertiary care.

Several limitations should be acknowledged. First, high attrition is inherent to digital health studies and may obscure later transitions. Engagement remains a major challenge in mobile health interventions.^41^ Nevertheless, sensitivity analyses using multiple methods (multiple imputation by chained equations, IPCW, and joint model) yielded consistent results, supporting the robustness of the findings and suggesting that stability is not an artifact of dropout.

Second, the 6-month follow-up period may be considered short relative to the chronic nature of pain. This choice was driven by attrition constraints, as longer follow-up would substantially reduce sample size and data completeness. As a result, longer-term trajectory patterns may not be captured. To address this limitation, a larger number of patients must be included to ensure a minimum analyzable sample size after 1 year or several years of follow-up, despite high attrition.

Third, pain intensity was not analyzed as a primary outcome. Although commonly used, it is increasingly recognized as an incomplete proxy for the chronic pain experience.^20^ Pain intensity often correlates poorly with functional disability and quality of life, particularly in chronic conditions.^19^ The interrelated dynamics of pain, physical impact, sleep, and mood justify the use of a multidimensional approach aligned with the biopsychosocial model. Nevertheless, the weekly measures for bodily comfort, sleep, and mood, while conceptually derived from validated instruments (PROMIS), have not been independently validated as standalone psychometric tools. This limits formal comparisons with standardized outcome measures used in other chronic pain studies.

Fourth, treatment changes occurring during follow-up were not systematically captured as time-varying covariates. As treatment adjustments represent a potential driver of trajectory transitions, their absence from longitudinal models may limit causal interpretation of the identified patterns.

A fifth limitation relates to the potential instability of clustering methods. GMM results can vary depending on model initialization, sample variability, and parameter selection. This is particularly relevant with high-dimensional behavioral data. However, the consistency of clustering results across multiple indicators and analytical approaches supports the robustness of the identified trajectories. Nonetheless, replication in larger cohorts is needed.

Sixth, the study population was limited to tertiary care patients, who often have long-standing and refractory pain. The high level of stability observed may, therefore, reflect entrenched clinical states. Studies in primary care or community settings may reveal more heterogeneous and potentially more predictable trajectories.^2,34^

## Conclusions

In this cohort study of 1477 adults with chronic noncancer pain followed up via weekly smartphone assessments over 6 months in French tertiary pain clinics, trajectories of bodily comfort, sleep, and mood were predominantly stable, with 4.1% of patients improving and 10.2% worsening. Long-term trajectory was not estimated by baseline characteristics. These findings support recognizing stability as a meaningful clinical state in tertiary care, with continuous digital monitoring and individualized deviation detection representing more actionable strategies than baseline-only prognostic stratification. Future research should explore hybrid models integrating early longitudinal signals with baseline variables to improve prediction of upcoming transitions.^42^

## Notes

Supplement 1. eMethods. eReferences eFigure 1. Overall methodological pipeline of the study eFigure 2. Participant retention over time eFigure 3. Longitudinal trajectories of patient-reported outcomes eFigure 4. Trajectory dynamics prior to study disengagement eFigure 5. Latent trajectory classes identified using K-means based on baseline features and PCA-transformed BLUPs eFigure 6. K-means cluster trajectories on raw weekly non-aggregated data across five missing data thresholds and imputation strategies eTable 1. Baseline characteristics of the eDOL cohort and analytical sample eTable 2. Comparison of adherent and non-adherent patients eTable 3. Statistical Performance and Selection Criteria for Multivariable Gaussian Mixture Models eTable 4. Performance metrics of predictive models for trajectory class membership

Supplement 2. Data Sharing Statement
