# Supplementary material for: Digital Monitoring and Chronic Pain Trajectories in Patients Receiving Tertiary Pain Care
Source: JAMA Netw Open. 2026 Aug 4;9(8):e2627217. doi: 10.1001/jamanetworkopen.2026.27217 (PMC13439428; doi:10.1001/jamanetworkopen.2026.27217)
Supplement: Supplementary file 1 [file jamanetwopen-e2627217-s001.pdf]

Supplemental Online Content

Kerckhove N, Soubeiga A, Phalip J, et al. Digital monitoring and chronic pain trajectories in patients receiving tertiary pain care. *JAMA Netw Open*. 2026;9(8):e2627217.
doi:10.1001/jamanetworkopen.2026.27217

eMethods.

eReferences

eFigure 1. Overall methodological pipeline of the study

eFigure 2. Participant retention over time

eFigure 3. Longitudinal trajectories of patient-reported outcomes

eFigure 4. Trajectory dynamics prior to study disengagement

eFigure 5. Latent trajectory classes identified using K-means based on baseline features and PCA-transformed BLUPs

eFigure 6. K-means cluster trajectories on raw weekly non-aggregated data across five missing data thresholds and imputation strategies

eTable 1. Baseline characteristics of the eDOL cohort and analytical sample

eTable 2. Comparison of adherent and non-adherent patients

eTable 3. Statistical Performance and Selection Criteria for Multivariable Gaussian Mixture Models

eTable 4. Performance metrics of predictive models for trajectory class membership

This supplemental material has been provided by the authors to give readers additional information about their work.

© 2026 Kerckhove N et al. *JAMA Network Open*.

eMethods

eDOL tool

All data were collected using the eDOL digital health tool, which includes a smartphone app for patients that allows self-questionnaires and assessments to be completed for semiological monitoring (pain, anxiety, sleep quality), and a web interface for physicians, to allow them to graphically visualize the summary of data provided by their patients for clinical and therapeutic monitoring. Patients completed questionnaires and weekly assessments. The questionnaires were divided into general questionnaires that were systematically filled in once only (sociodemographic, lifestyle and professional data, Pain Beliefs and Perceptions Inventory; Evaluation of level of precariousness; Injustice Experience Questionnaire; Maslach Burn-out Inventory; Toronto Alexithymia Scale; Life Orientation Test-Revised; Belief in a just world; Job Content Questionnaire; Big Five Inventory) and questionnaires, assessing symptoms, comorbidities, and psychological and physiological states related to chronic pain, that were completed quarterly (Brief Pain Inventory; Medical Outcomes Study Sleep Scale) and, according to duration of follow-up, half-yearly (Tampa Scale of Kinesiophobia; Pain Catastrophizing Scale; Fear-avoidance beliefs; EQ-5D-3L; Hospital Anxiety Depression Scale; Satisfaction With Life Scale; Subjective Cognitive Complaints). Some questionnaires were specific to a type of chronic pain (Neuropathic Pain Scale Inventory; Western Ontario and McMaster Universities; Rheumatoid Arthritis Impact of Disease; Roland Morris Disability Questionnaire; Irritable Bowel Severity Scoring System; Fibromyalgia Impact Questionnaire; Headache Impact Test). Follow-up of patients (daily monitoring of various objective and subjective parameters related to the pathology), using assessments, was also integrated in the app, which allowed us to monitor the evolution of patients' pain and its repercussions. Assessments were in the form of an 11-point numeric rating scale (from 0 to 10), assessing the intensity of pain (average, minimum, or maximum intensity), anxiety, fatigue, and the quality of sleep, morale, bodily comfort were assessed weekly.

For physicians, the eDOL internet platform included a simple and ergonomic dashboard which allowed the physician to find all of their patients included in the study, with the following tabs:

© 2026 Kerckhove N et al. *JAMA Network Open*.

(1) Management, in which all of the medical records completed by the physician could be found (history, pain diagnosis, initial characterization, next appointment, consultation sheets and treatment sheets); (2) Health Measures, which showed a graphic display of the real-life follow-up of all the weekly assessments; and (3) Questionnaires, which showed all the questionnaires completed by the patients (display of questionnaire scores and answers to all the questions). The eDOL platform enabled physicians to complete medical elements during consultation visits with various medical form (diagnosis, current treatments, examination results). The physicians could also activate new questionnaires to be filled in by their patients, either to complete the characterization (eg, specific questionnaires for pain diagnosis) or to evaluate other criteria (eg, evaluation of the Patients' Global Impression of Change after the introduction of a new treatment). Diagnostic questionnaires (Posttraumatic stress disorder Checklist; Neuropathic pain 4; Fibromyalgia Rapid Screening Tool), reminders of the criteria for diagnoses (ROME IV for irritable bowel syndrome; Widespread pain index and Symptom severity scale of American college of rheumatology for fibromyalgia; Neuropathic Pain IASP Special Interest Groups for neuropathic pain), and screening tools for opioid misuse (Prescription Opioid Misuse Index and Opioid Risk Tool) were also at their disposal

Study design and participants

This study is a secondary analysis of the eDOL mHealth cohort[1], a web platform and mobile application designed for the longitudinal follow-up of patients with chronic non-cancer pain. Participants began follow-up in pain clinic were recruited in real-world settings across multiple pain clinics in France (from September 2019 to September 2025) and followed via the eDOL web platform and mobile app for up to 28 weeks. The eDOL tool integrates a wide range of sociodemographic and medical data. All patients with a theoretical follow-up of at least 6 months with eDOL tool and with baseline data in the first month of follow-up were included.

The study was approved by the research ethics committee and the French Data Protection Authority. The study has been registered at ClinicalTrials.gov (NCT04880096). The study was

© 2026 Kerckhove N et al. *JAMA Network Open*.

conducted in accordance with French laws and regulations on research on human beings and data protection, and in accordance with the Declaration of Helsinki.

Data collection and outcomes

We focused our trajectory analysis on three multidimensional, completed weekly on a 11-point numeric rating scale (0 = poor quality and 10 = high quality), patient-reported outcome measures (PROMs): bodily comfort, sleep, and mood, which better capture the dynamic burden of chronic pain.

Data were collected weekly over a maximum follow-up period of 28 weeks. Weekly data were monthly aggregated (computed as the median score within each month) in order to reduce measurement noise and short-term fluctuations inherent to PROMs. This aggregation strategy also reduces the influence of sporadic missing reports and has been recommended when analyzing high-frequency digital health data to stabilize trajectory estimation without eliminating clinically meaningful changes[2,3].

Study objectives

The study aimed to: (i) characterize the stability and variability of patient-reported outcomes across bodily comfort, sleep, and mood domains; (ii) identify latent trajectory classes using advanced clustering techniques; and (iii) evaluate the predictive capacity of baseline variables using machine learning, accounting rigorously for missing data and informative attrition. Overall methodological pipeline to achieve the objectives is illustrated in **Figure 1**.

Longitudinal modeling and attrition management

Attrition was defined as the absence of any recorded data during the 6th month of follow-up (weeks 24 to 28). Two established approaches were applied to correct for attrition bias, according to missing at random (MAR) or missing not at random (MNAR) mechanisms. First, IPCW models were estimated using a logistic regression model to predict the probability of

© 2026 Kerckhove N et al. *JAMA Network Open*.

being observed at the target time point, based on baseline covariates (age, sex, pain duration, and baseline PROMIS interference score)[4,5]. Second, joint longitudinal-survival models were fitted to account for bias due to MNAR[6,7]. The longitudinal submodel was a linear mixed model (LMM) with random intercepts and slopes for each patient, while the survival submodel employed a Cox model describing the time to dropout, allowing estimation of the effect of individual score trajectories on the probability of censoring[8]. These models were used to link the longitudinal process (scores) and the event process (attrition).

Missing data imputation and sensitivity analyses

To assess the robustness of the observed trajectories against missing data patterns, we conducted a sensitivity analysis using Multiple Imputation by Chained Equations (MICE) via the *mice* R package[9,10]. The imputation model included all longitudinal observations to account for the temporal structure of the data. Missing values for the three longitudinal outcomes (bodily comfort, sleep, and mood) were imputed using Predictive Mean Matching (PMM), which preserves the non-normal distributional properties and the bounded nature of the original scales[11]. We generated 50 imputed datasets (m=50) with 10 iterations to ensure convergence[12,13]. Consistent with best practices for sensitivity analysis, we verified that the distribution of latent trajectories remained stable across imputed datasets compared to the primary analysis (observed data with IPCW). This approach ensured that the identified latent classes and clinical transition patterns were not artifacts of the missing data management strategy.

Intra-individual dynamics were also assessed using the time-in-state approach, which estimates the proportion of time each patient spent in clinically defined states (weekly status measurement) of improvement, stagnation, or deterioration[14]. This method treats dynamics as discrete states and quantifies clinical stability rather than continuous variation alone. States were determined with at least -1.5 points in score for deterioration or at least +1.5 points in score for improvement. This threshold was chosen in accordance with the work of Kovacs et

© 2026 Kerckhove N et al. *JAMA Network Open*.

al.[15] and Mannerkorpi et al.[16], suggesting that changes of less than 1.5 are clinically irrelevant in chronic musculoskeletal and nociplastic pains, and represents a conservative compromise between the minimum detectable change and the major clinical improvement typically set at 2 points[17].

Latent class analysis

To characterize individual trajectories, two types of features were derived: (i) baseline PROM values and (ii) subject-specific random effects (BLUPs) estimated from mixed-effects models. To reduce dimensionality and account for potential correlations among BLUPs, a principal component analysis (PCA) was applied prior to clustering. Trajectory clustering was then performed using two complementary approaches: (i) K-means clustering applied to baseline features and to PCA-transformed BLUPs, and (ii) Gaussian Mixture Models (GMM) applied to monthly aggregated PROMs and after IPCW and joint model approaches.

GMM applied to longitudinal PROM summaries provided more distinct and interpretable trajectory groups (see **Results section**), and was therefore retained as the primary method for trajectory analysis.

Unlike polynomial-based Group-Based Trajectory Models (GBTM), GMM treats each patient's six-month trajectory as a single multidimensional vector, allowing for flexible, non-linear patterns typical of real-world mHealth data[18]. Specifically, the clustering was performed in the 6-dimensional space formed by the monthly medians (M1 to M6) for each outcome. This non-parametric approach is particularly suited for real-world mHealth data, as it allows for the discovery of stable or unconventional patterns without imposing a rigid growth structure.

Compared to k-means clustering, GMM offers several advantages for modeling complex health data. While k-means assumes spherical clusters with equal variance and assigns each observation to a single cluster through hard partitioning, GMM uses a probabilistic framework that allows clusters to have different shapes, sizes, and covariance structures (i.e., capturing correlations between months). This flexibility is crucial for datasets where clinical trajectories

often overlap. Furthermore, GMM provides soft assignments, where each observation has a probability of belonging to each cluster. This captures the inherent uncertainty in behavioral health states and individual variability more realistically than deterministic methods[19,20].

To determine the optimal number of latent trajectories, we estimated models ranging from 1 to 4 classes. Model selection was based on the Bayesian Information Criterion (BIC), Sample-Size Adjusted BIC (SABIC), and relative entropy (value closer to 1), and clinical interpretability of the trajectories. The internal validity of the classification was rigorously assessed using the Odds of Correct Classification (OCC), with a threshold strictly superior to 5 required to confirm that classes were distinct clinical entities rather than statistical artifacts[21]. Statistical uncertainty for latent class trajectories was estimated using a Monte Carlo approach with 2,000 realizations of the model parameters to compute 95% confidence intervals for each predicted outcome.

To characterize the clinical profiles of the identified patient trajectories, we employed the CATDES (Characterization of Categories) procedure, integrated within the Hierarchical Clustering on Principal Components (HCPC) framework[22]. The primary objective of this method is to identify the specific variables (continuous) and categories (nominal) that significantly discriminate each cluster from the overall study population. The significance of each variable's contribution to a cluster is assessed using the V-Test (Test-Value). This statistic measures the deviation between the mean of a variable within a specific class and the overall population mean. Under the null hypothesis (H0), which assumes that the individuals in a cluster are a random sample from the total population, the V-Test follows a standard normal distribution. A V-Test > 1.96 indicates a significant difference at the alpha = 0.05, and a positive V-test (V > 0) signifies that the variable or category is over-represented or significantly higher in the class compared to the overall population.

Predictive modeling and machine learning

Finally, predictive models for trajectory membership were tested using machine learning algorithms, including random forest (RF) (and balanced RF), Gradient Boosting (XGBoost),

© 2026 Kerckhove N et al. *JAMA Network Open*.

support vector machines (SVM), K-nearest neighbors (KNN), CatBoost and multinomial logistic regression based solely on sociodemographic and baseline clinical variables and using the caret and randomForest R packages. Hyperparameters, such as the number of trees (range 100–1000) and maximum depth for RF, were optimized via grid search. Model performance was evaluated using 10-fold cross-validation to prevent overfitting. Final predictive performance was reported using metrics robust to imbalance, specifically Cohen's Kappa, Balanced accuracy, and the area under the Precision-Recall curve, rather than simple accuracy[23,24].

© 2026 Kerckhove N et al. *JAMA Network Open*.

eReferences

1. Kerckhove N, Delage N, Cambier S, et al. eDOL mHealth App and Web Platform for Self-monitoring and Medical Follow-up of Patients With Chronic Pain: Observational Feasibility Study. *JMIR Form Res*. 2022;6(3):e30052. doi:10.2196/30052

2. Schwartz JE, Stone AA. Strategies for analyzing ecological momentary assessment data. *Health Psychol*. 1998;17(1):6-16. doi:10.1037//0278-6133.17.1.6

3. Intensive Longitudinal Methods: An Introduction to Diary and Experience Sampling Research. Guilford Press. Accessed March 18, 2026. https://www.guilford.com/books/Intensive-Longitudinal-Methods/Bolger-Laurenceau/9781462506781?srsltid=AfmBOopjUqrSi3lQP6GcKH5GjfUgWqwI_SOiu_bDTN87WBBx_VNcooGL

4. Robins JM, Finkelstein DM. Correcting for Noncompliance and Dependent Censoring in an AIDS Clinical Trial with Inverse Probability of Censoring Weighted (IPCW) Log-Rank Tests. *Biometrics*. 2000;56(3):779-788. doi:10.1111/j.0006-341X.2000.00779.x

5. Dong G, Mao L, Huang B, et al. The inverse-probability-of-censoring weighting (IPCW) adjusted win ratio statistic: an unbiased estimator in the presence of right censoring. *J Biopharm Stat*. 2020;30(5):882-899. doi:10.1080/10543406.2020.1757692

6. Daniels, M.J. and Hogan, J.W. (2008) Missing Data in Longitudinal Studies Strategies for Bayesian Modeling and Sensitivity Analysis. Taylor & Francis, Boca Raton, FL. - References - Scientific Research Publishing. Accessed March 18, 2026. https://www.scirp.org/reference/referencespapers?referenceid=2020026

7. Rizopoulos D. *Joint Models for Longitudinal and Time-to-Event Data: With Applications in R*. Chapman and Hall/CRC; 2012. doi:10.1201/b12208

8. *Linear Mixed Models for Longitudinal Data | Geert Verbeke | Springer*. Accessed February 9, 2017. http://www.springer.com/la/book/9781441902993

9. Buuren S van, Groothuis-Oudshoorn K. mice: Multivariate Imputation by Chained Equations in R. *Journal of Statistical Software*. 2011;45:1-67. doi:10.18637/jss.v045.i03

10. White IR, Royston P, Wood AM. Multiple imputation using chained equations: Issues and guidance for practice. *Stat Med*. 2011;30(4):377-399. doi:10.1002/sim.4067

11. Vink G, Frank LE, Pannekoek J, Buuren S. Predictive mean matching imputation of semicontinuous variables. *Statistica Neerlandica*. 2014;68(1):61-90.

12. Graham JW, Olchowski AE, Gilreath TD. How many imputations are really needed? Some practical clarifications of multiple imputation theory. *Prev Sci*. 2007;8(3):206-213. doi:10.1007/s11121-007-0070-9

13. Sterne JAC, White IR, Carlin JB, et al. Multiple imputation for missing data in epidemiological and clinical research: potential and pitfalls. *BMJ*. 2009;338:b2393. doi:10.1136/bmj.b2393

14. Kim M, Yang J, Ahn WY, Choi HJ. Machine Learning Analysis to Identify Digital Behavioral Phenotypes for Engagement and Health Outcome Efficacy of an mHealth Intervention for Obesity: Randomized Controlled Trial. *Journal of Medical Internet Research*. 2021;23(6):e27218. doi:10.2196/27218

© 2026 Kerckhove N et al. *JAMA Network Open*.

15. Kovacs FM, Abraira V, Royuela A, et al. Minimum detectable and minimal clinically important changes for pain in patients with nonspecific neck pain. *BMC Musculoskelet Disord*. 2008;9:43. doi:10.1186/1471-2474-9-43

16. Mannerkorpi K, Nordeman L, Cider Å, Jonsson G. Does moderate-to-high intensity Nordic walking improve functional capacity and pain in fibromyalgia? A prospective randomized controlled trial. *Arthritis Res Ther*. 2010;12(5):R189. doi:10.1186/ar3159

17. Dworkin RH, Turk DC, Wyrwich KW, et al. Interpreting the clinical importance of treatment outcomes in chronic pain clinical trials: IMMPACT recommendations. *J Pain*. 2008;9(2):105-121. doi:10.1016/j.jpain.2007.09.005

18. Proust-Lima C, Philipps V, Liquet B. Estimation of Extended Mixed Models Using Latent Classes and Latent Processes: The R Package lcmm. *Journal of Statistical Software*. 2017;78:1-56. doi:10.18637/jss.v078.i02

19. *Pattern Recognition and Machine Learning*. Accessed March 18, 2026. https://link.springer.com/book/9780387310732

20. Murphy KP. *Machine Learning: A Probabilistic Perspective*. MIT Press; 2012.

21. McNicholas PD. *Mixture Model-Based Classification*. Chapman and Hall/CRC; 2016. doi:10.1201/9781315373577

22. Husson F, Le S, Pagès J. *Exploratory Multivariate Analysis by Example Using R*. 2nd ed. Chapman and Hall/CRC; 2017. doi:10.1201/b21874

23. Chawla NV, Bowyer KW, Hall LO, Kegelmeyer WP. SMOTE: Synthetic Minority Over-sampling Technique. *jair*. 2002;16:321-357. doi:10.1613/jair.953

24. Saito T, Rehmsmeier M. The Precision-Recall Plot Is More Informative than the ROC Plot When Evaluating Binary Classifiers on Imbalanced Datasets. *PLOS ONE*. 2015;10(3):e0118432. doi:10.1371/journal.pone.0118432

© 2026 Kerckhove N et al. *JAMA Network Open*.

eFigure 1: Overall methodological pipeline of the study. Workflow illustrating the analytical strategy used to investigate chronic pain trajectories in the eDOL cohort. The pipeline includes data preprocessing, management of informative attrition (IPCW and joint models), trajectory characterization using time-in-state analyses and linear mixed models, identification of latent trajectory classes using Gaussian mixture models, and prediction of trajectory membership using machine learning algorithms.

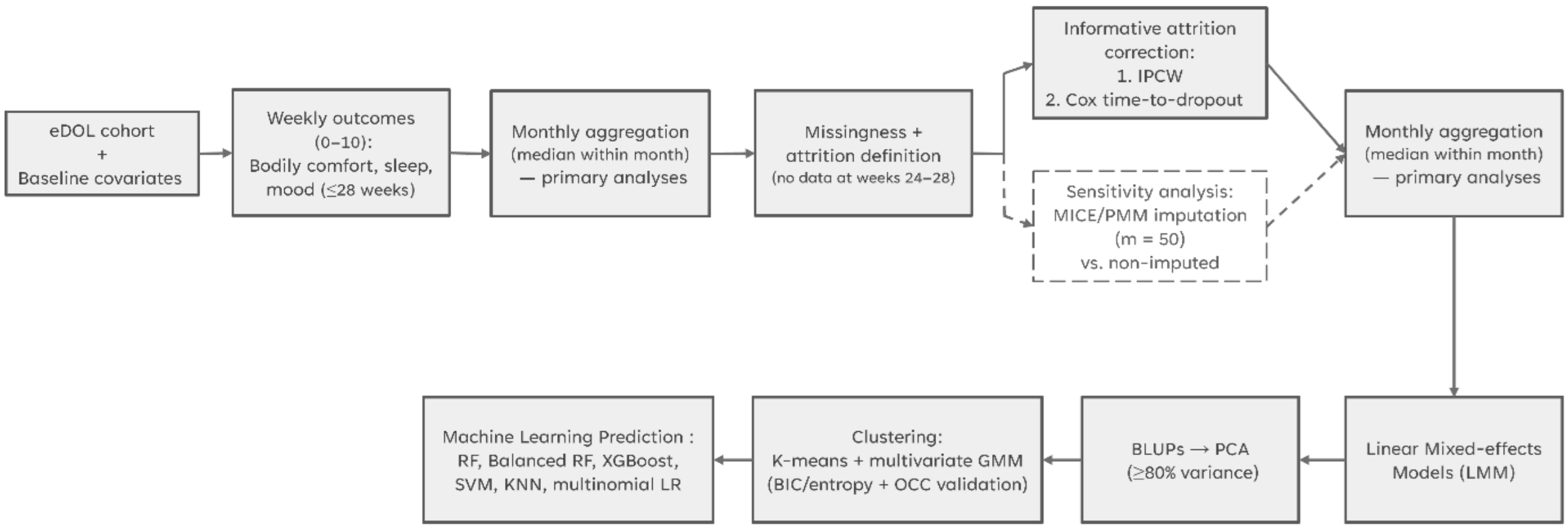

© 2026 Kerckhove N et al. *JAMA Network Open*.

eFigure 2: Participant retention over time. Kaplan-Meier curve showing the probability of remaining active in the eDOL monitoring system over the 6-month follow-up period

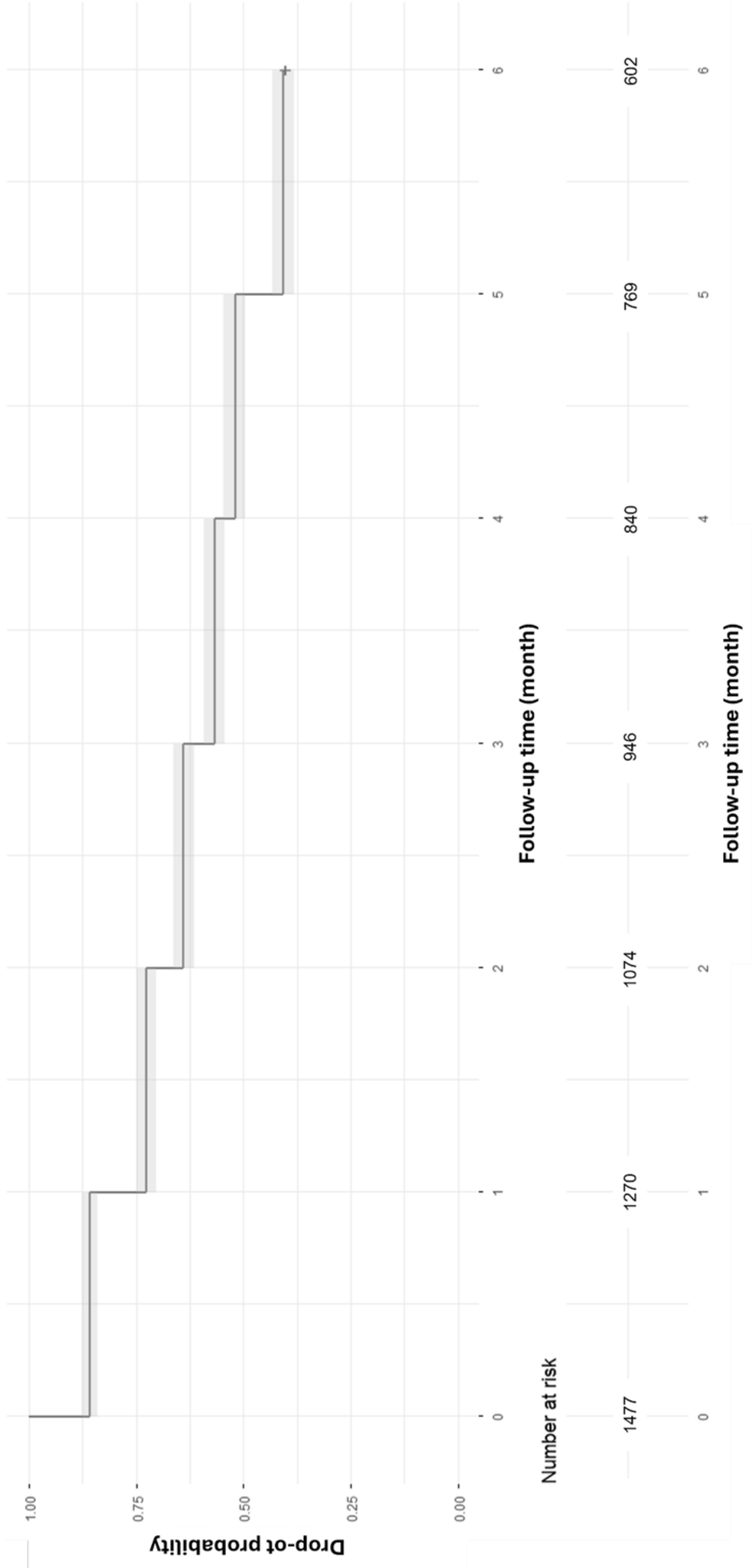

© 2026 Kerckhove N et al. *JAMA Network Open*.

eFigure 3: Longitudinal trajectories of patient-reported outcomes.

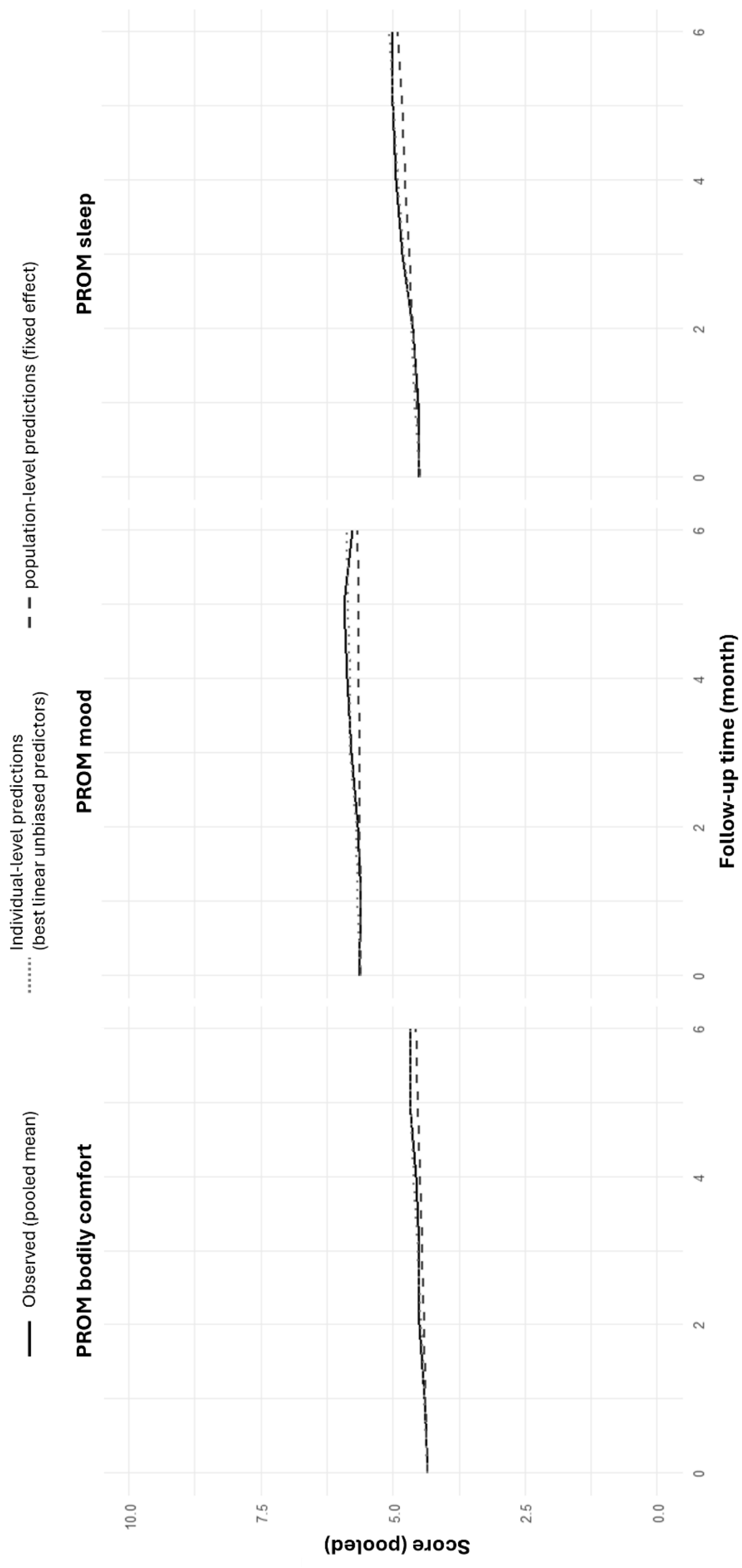

Average trajectories of bodily comfort, sleep, and mood over time estimated using linear mixed-effects models. Solid lines represent observed pooled mean trajectories. Dotted lines represent individual-level predictions derived from best linear unbiased predictors, and dashed lines

© 2026 Kerckhove N et al. *JAMA Network Open*.

represent population-level predictions based on fixed effects. Models incorporate inverse probability of censoring weighting and joint modeling to account for informative attrition.

© 2026 Kerckhove N et al. *JAMA Network Open*.

eFigure 4: Trajectory dynamics prior to study disengagement. Weekly evolution of patient states (proportion of patients per week) classified as improvement (gray), decline (dark gray), or stagnation (light gray) across different missing-data scenarios and imputation strategies: < 10% miss (No Imp) = patients with less than 10% missing data, no imputation; < 25% miss (No Imp) = patients with less than 25% missing data, no imputation; < 25% miss (With Imp) = patients with less than 25% missing data, with imputation; < 50% miss (With Imp) = patients with less than 50% missing data, with imputation.

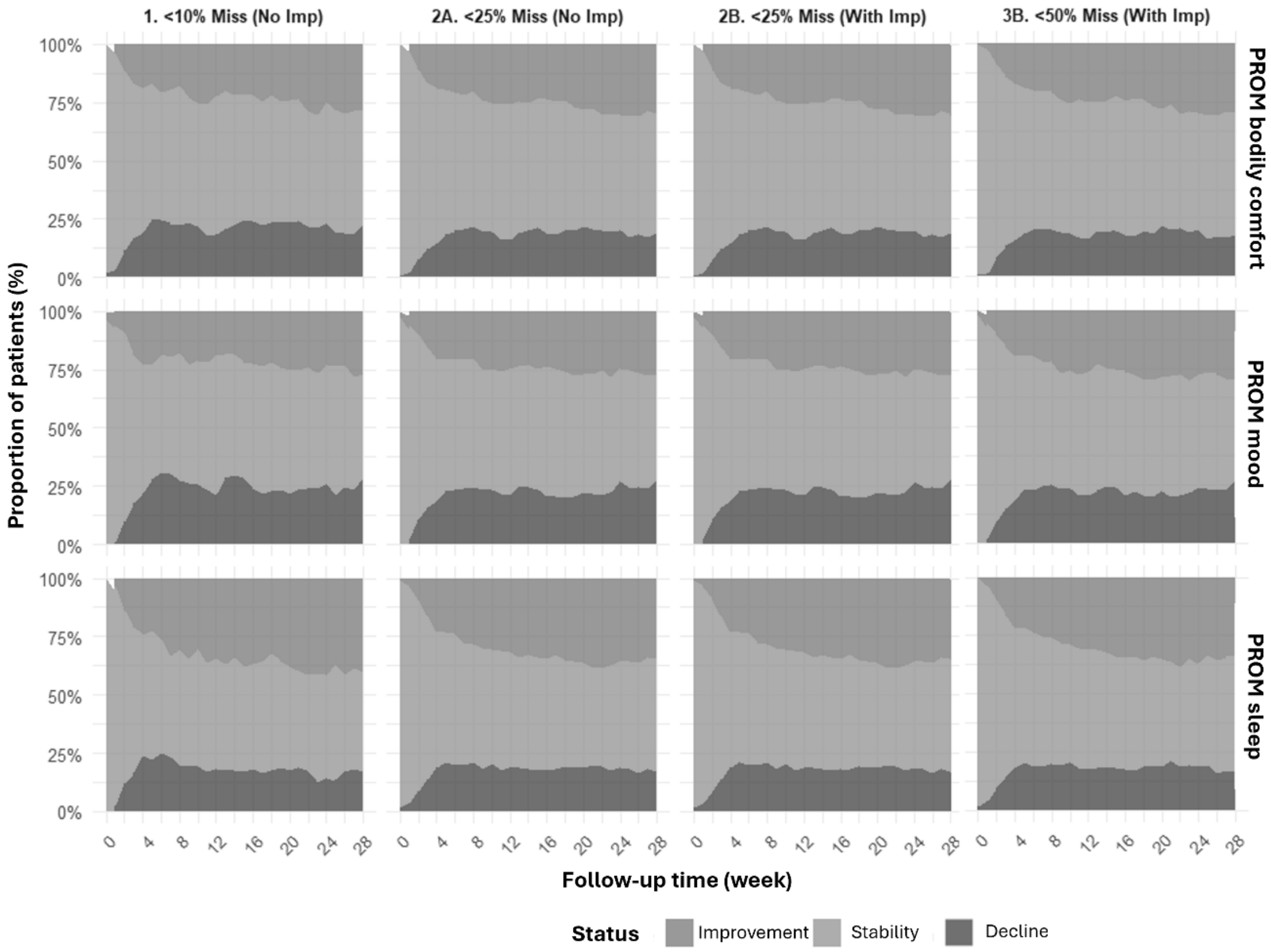

© 2026 Kerckhove N et al. *JAMA Network Open*.

eFigure 5: Latent trajectory classes identified using K-means based on baseline features and PCA-transformed BLUPs. A. Observed PROMs values and model-estimated trajectories for the three latent classes identified by the multivariate K-means based on baseline features analysis. Thick lines represent model-estimated mean trajectories for each class, and the thin lines represent trajectories of each individual. B. Projection of individual-specific random effects (BLUPs) onto the first two principal components. Each point corresponds to a subject, with geometric shapes indicating cluster assignment (three classes). PCA was applied to reduce dimensionality and mitigate collinearity among BLUPs prior to clustering.

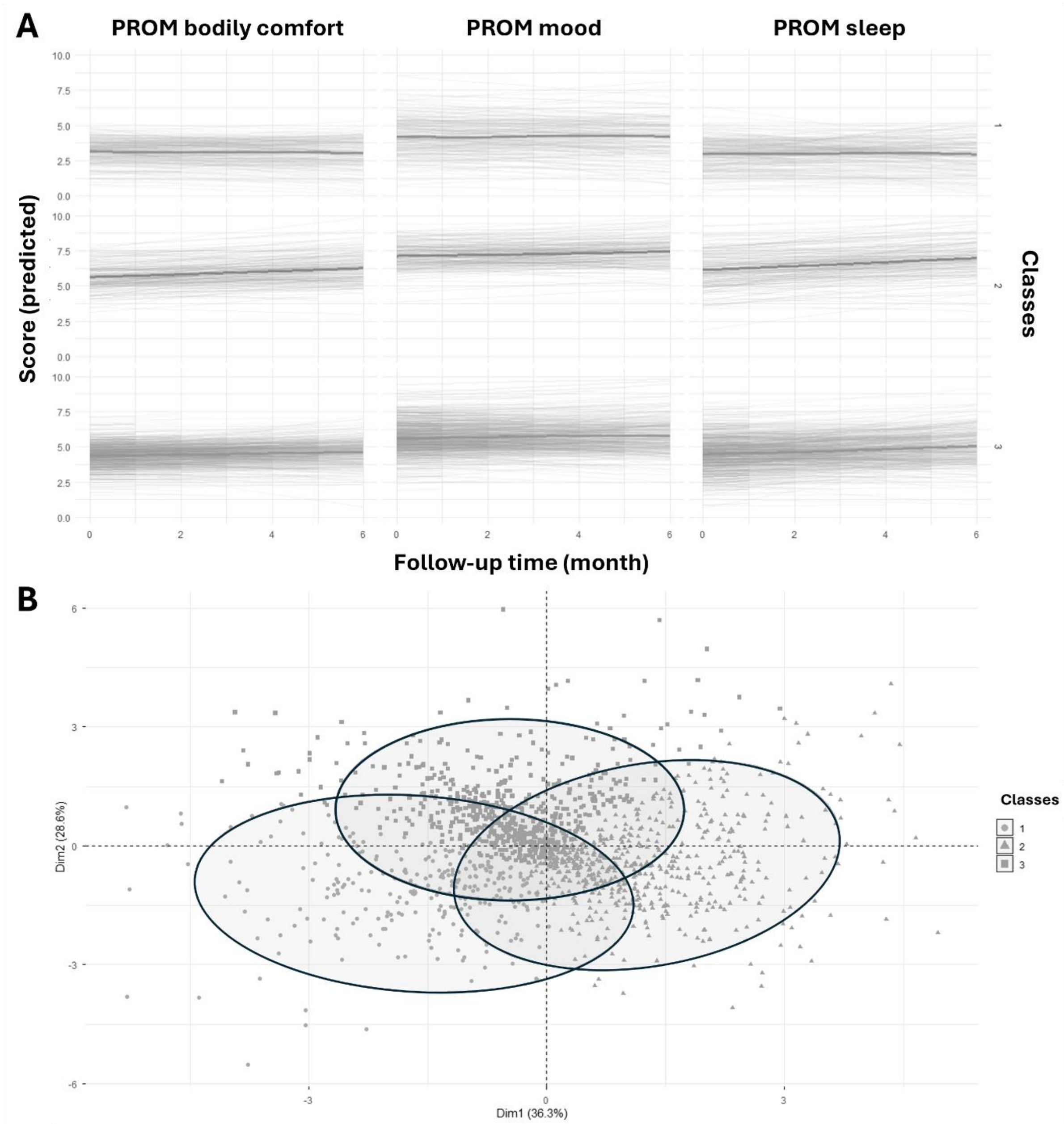

© 2026 Kerckhove N et al. *JAMA Network Open*.

eFigure 6: K-means cluster trajectories on raw weekly non-aggregated data across five missing data thresholds and imputation strategies. Observed PROMs values and model-estimated trajectories for the three latent classes (dark grey; light grey or grey) identified by the multivariate K-means based on baseline features analysis across different missing-data scenarios and imputation strategies: a. <= 10% missing data and no imputation; b. > 10% and <= 25% missing data and imputation; c. > 25% and <= 50% missing data and imputation; > 50% and <= 75% missing data and no imputation; > 75% missing data and imputation.

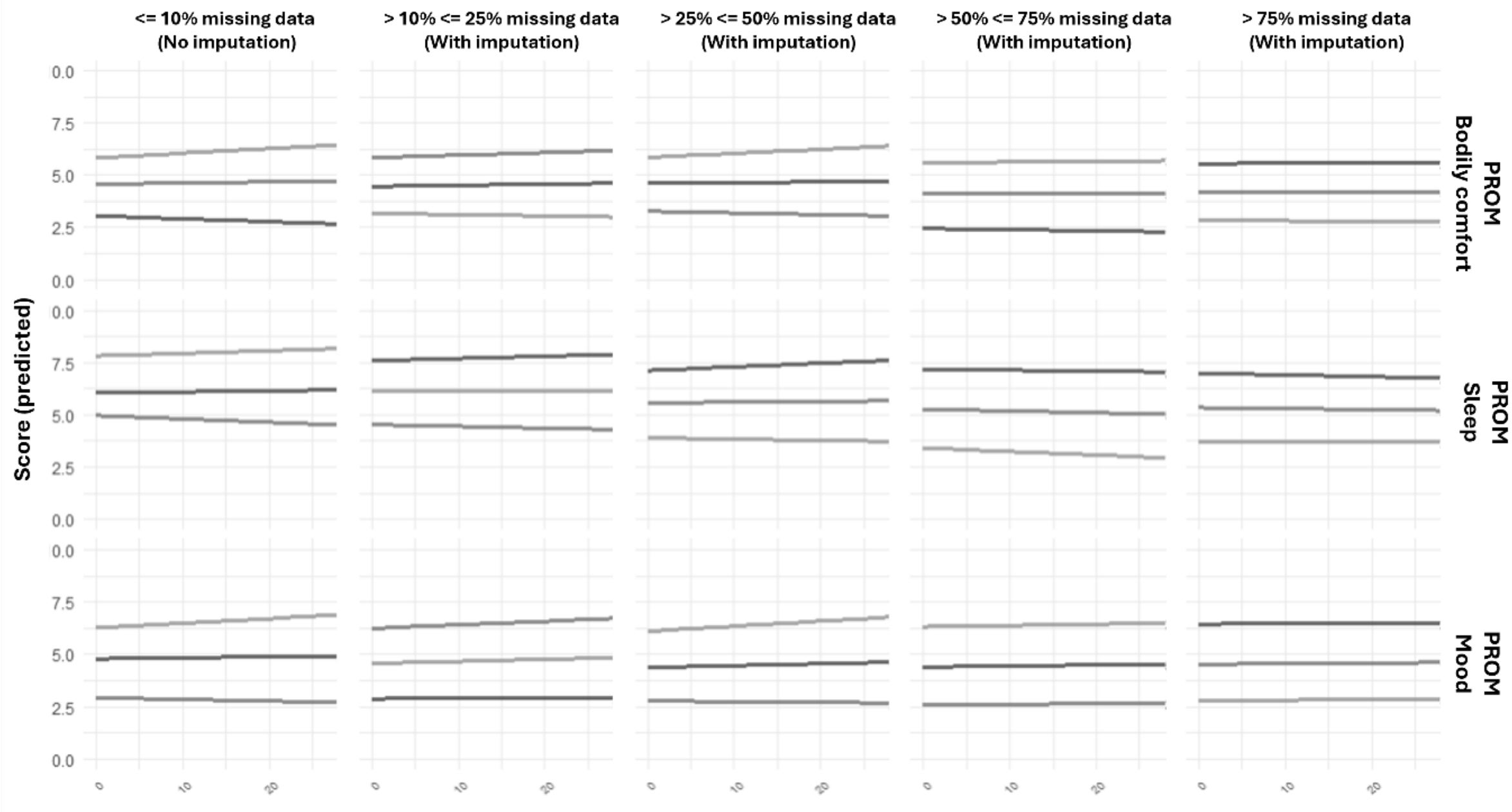

© 2026 Kerckhove N et al. *JAMA Network Open*.

eTable 1: Baseline characteristics of the eDOL cohort and analytical sample. Sociodemographic, lifestyle, and clinical characteristics of participants at study entry in the full eDOL cohort (n=2207) and in the analytical sample included in trajectory analyses (n=1477). Continuous variables are presented as mean (SD) and categorical variables as counts with percentages and 95% confidence intervals.

Variables	Patients eDOL cohort (n = 2207)	Patients analyzed (n = 1477)
**Sex and Body Mass Index**		
Female, n (%) [95% CI]	1,773 (80.5%) [78.7 to 82.1]	1,213 (82.1%) [77.9 to 86.3]
Male, n (%) [95% CI]	429 (19.5%) [17.8 to 21.2]	262 (17.7%) [16.8 to 18.6]
BMI, mean (SD)	27.4 (7.0)	27.4 (7.0)
BMI > 25, n (%) [95% CI]	1,238 (56.1%) [54.0 to 58.2]	822 (55.6%) [53.1 to 58.2]
**Age class, n (%) [95% CI]**		
18 to 24 years	96 (4.4%) [3.5 to 5.3]	65 (4.4%) [4.2 to 4.6]
25 to 34 years	271 (12.3%) [10.9 to 13.7]	180 (12.2%) [11.6 to 12.8]
35 to 49 years	850 (38.5%) [36.5 to 40.6]	577 (39.1%) [37.1 to 41.1]
50 to 64 years	802 (36.4%) [34.4 to 38.4]	557 (37.7%) [35.8 to 39.6]
≥65 years	186 (8.4%) [7.3 to 9.7]	98 (6.6%) [6.3 to 7.0]
**Family situation**		
In a relationship, n (%) [95% CI]	1,280 (66.7%) [64.5 to 68.8]	1,014 (68.7%) [65.2 to 72.2]
Single, n (%) [95% CI]	639 (33.3%) [31.2 to 35.5]	463 (31.3%) [29.7 to 32.9]
Number of children, mean (SD)	1.2 (1.2)	1.2 (1.2)
**Level of education, n (%) [95% CI]**		
Below high school diploma	545 (28.4%) [26.4 to 30.5]	410 (27.8%) [26.3 to 29.2]
High school diploma to Bachelor's degree	999 (52.1%) [49.8 to 54.3]	780 (52.8%) [50.1 to 55.5]
Above Bachelor's degree	375 (19.5%) [17.8 to 21.4]	287 (19.4%) [18.4 to 20.4]
**Employment status, n (%) [95% CI]**		
Employed	1,125 (58.6%) [56.4 to 60.8]	869 (58.8%) [55.8 to 61.8]
Unemployed	794 (41.4%) [39.2 to 43.6]	608 (41.2%) [39.1 to 43.3]
Disability allowance	181 (8.2%) [7.1 to 9.4]	138 (9.3%) [8.9 to 9.8]
Work to related disability	37 (1.7%) [1.2 to 2.3]	31 (2.1%) [2.0 to 2.2]
Non to work to related disability	415 (18.8%) [17.2 to 20.5]	333 (22.5%) [21.4 to 23.7]
Adapted work position	98 (4.4%) [3.6 to 5.4]	76 (5.1%) [4.9 to 5.4]
Recognition of disabled worker status	271 (12.3%) [10.9 to 13.7]	226 (15.3%) [14.5 to 16.1]
Sick leave related to pain	430 (19.5%) [17.9 to 21.2]	322 (21.8%) [20.7 to 22.9]
Sick leave unrelated to pain	55 (2.5%) [1.9 to 3.2]	44 (3.0%) [2.8 to 3.1]
**Tobacco and alcohol consumption, n (%) [95% CI]**		
Former smoker	1,107 (60.7%) [58.4 to 62.9]	884 (59.9%) [56.8 to 62.9]
Current smoker	451 (24.7%) [22.8 to 26.8]	360 (24.4%) [23.1 to 25.6]
Never smoked	266 (14.6%) [13.0 to 16.3]	226 (15.3%) [14.5 to 16.1]
No alcohol or less than once per month	733 (40.2%) [37.9 to 42.5]	581 (39.3%) [37.3 to 41.3]
Alcohol about once per month	437 (24.0%) [22.0 to 26.0]	372 (25.2%) [23.9 to 26.5]
Alcohol 2 to 4 times per month	447 (24.5%) [22.5 to 26.5]	357 (24.2%) [22.9 to 25.4]
Alcohol 2 to 3 times per week	148 (8.1%) [6.9 to 9.5]	113 (7.7%) [7.3 to 8.0]
Alcohol at least 4 times per week	59 (3.2%) [2.5 to 4.2]	47 (3.2%) [3.0 to 3.3]
**Chronic pain duration, n (%) [95% CI]**		
3 to 5 months	13 (0.7%) [0.4 to 1.2]	11 (0.7%) [0.7 to 0.8]
6 to 11 months	96 (5.3%) [4.3 to 6.4]	73 (5.0%) [4.7 to 5.2]

© 2026 Kerckhove N et al. *JAMA Network Open*.

1 to 2 years	231 (12.7%) [11.2 to 14.3]	182 (12.4%) [11.7 to 13.0]
3 to 4 years	313 (17.2%) [15.5 to 19.0]	246 (16.7%) [15.9 to 17.6]
5 to 10 years	508 (27.9%) [25.8 to 30.0]	414 (28.2%) [26.7 to 29.6]
More than 10 years	663 (36.3%) [34.1 to 38.6]	544 (37.0%) [35.1 to 38.9]
**Chronic pain diagnosis**		
Number of chronic pain diagnoses, mean (SD)	1.2 (0.6)	1.3 (0.6)
Nociplastic, n (%) [95% CI]	760 (34.5%) [32.5 to 36.5]	548 (37.1%) [35.2 to 39.0]
Musculoskeletal, n (%) [95% CI]	477 (21.6%) [19.9 to 23.4]	313 (21.2%) [20.1 to 22.3]
Other, n (%) [95% CI]	343 (15.5%) [14.1 to 17.1]	230 (15.6%) [14.8 to 16.4]
Peripheral neuropathic, n (%) [95% CI]	310 (14.1%) [12.6 to 15.6]	204 (13.8%) [13.1 to 14.5]
Post to surgical or post to traumatic, n (%) [95% CI]	275 (12.5%) [11.1 to 13.9]	185 (12.5%) [11.9 to 13.2]
Headache, n (%) [95% CI]	265 (12.0%) [10.7 to 13.4]	177 (12.0%) [11.4 to 12.6]
Visceral, n (%) [95% CI]	203 (9.2%) [8.0 to 10.5]	140 (9.5%) [9.0 to 10.0]
Central neuropathic, n (%) [95% CI]	85 (3.9%) [3.1 to 4.7]	57 (3.9%) [3.7 to 4.1]
Post to cancer treatment, n (%) [95% CI]	39 (1.8%) [1.3 to 2.4]	23 (1.6%) [1.5 to 1.6]
**Chronic pain pharmacological treatments**		
Number of analgesic treatments, mean (SD)	2.1 (1.6)	2.3 (1.6)
Antidepressants recommended for the treatment of chronic pain, n (%) [95% CI]*	819 (37.1%) [35.1 to 39.2]	566 (38.3%) [36.4 to 40.3]
Paracetamol, n (%) [95% CI]	791 (35.9%) [33.9 to 37.9]	564 (38.2%) [36.2 to 40.1]
Other, n (%) [95% CI]	541 (24.5%) [22.7 to 26.4]	434 (29.4%) [27.9 to 30.9]
*Anticonvulsants recommended for the treatment of chronic pain**, n (%) [95% CI]*	449 (20.4%) [18.7 to 22.1]	321 (21.7%) [20.6 to 22.8]
Opioids, n (%) [95% CI]	393 (17.8%) [16.2 to 19.5]	276 (18.7%) [17.7 to 19.6]
NSAIDs, n (%) [95% CI]	305 (13.8%) [12.4 to 15.3]	202 (13.7%) [13.0 to 14.4]
Capsaicin, n (%) [95% CI]	243 (11.0%) [9.7 to 12.4]	151 (10.2%) [9.7 to 10.7]
Anesthetics, n (%) [95% CI]	226 (10.2%) [9.0 to 11.6]	157 (10.6%) [10.1 to 11.2]
Anxiolytics/hypnotics, n (%) [95% CI]	210 (9.5%) [8.3 to 10.8]	150 (10.2%) [9.6 to 10.7]
Others antidepressants, n (%) [95% CI]	192 (8.7%) [7.6 to 10.0]	142 (9.6%) [9.1 to 10.1]
Triptans (anti to migraine), n (%) [95% CI]	159 (7.2%) [6.2 to 8.4]	118 (8.0%) [7.6 to 8.4]
Nefopam, n (%) [95% CI]	143 (6.5%) [5.5 to 7.6]	103 (7.0%) [6.6 to 7.3]
Ketamine, n (%) [95% CI]	75 (3.4%) [2.7 to 4.2]	46 (3.1%) [3.0 to 3.3]
Botulinum toxin, n (%) [95% CI]	66 (3.0%) [2.3 to 3.8]	35 (2.4%) [2.2 to 2.5]
Others anticonvulsants, n (%) [95% CI]	59 (2.7%) [2.0 to 3.4]	35 (2.4%) [2.2 to 2.5]
Cannabinoids, n (%) [95% CI]	40 (1.8%) [1.3 to 2.5]	30 (2.0%) [1.9 to 2.1]
Others anti to inflammatory drugs, n (%) [95% CI]	26 (1.2%) [0.8 to 1.7]	23 (1.6%) [1.5 to 1.6]
Antirheumatic drugs, n (%) [95% CI]	1 (0.0%) [0.0 to 0.3]	1 (0.1%) [0.1 to 0.1]
**Chronic pain non to pharmacological treatments**		
Number of analgesic technics, mean (SD)	1.4 (1.0)	1.5 (1.0)
*Neuromodulation***, n (%) [95% CI]*	962 (43.6%) [41.5 to 45.7]	672 (45.5%) [43.2 to 47.8]
Physical and manual techniques, n (%) [95% CI]	940 (42.6%) [40.5 to 44.7]	675 (45.7%) [43.4 to 48.0]
Other, n (%) [95% CI]	515 (23.3%) [21.6 to 25.2]	368 (24.9%) [23.6 to 26.2]
Psychotherapeutic approaches, n (%) [95% CI]	371 (16.8%) [15.3 to 18.4]	275 (18.6%) [17.7 to 19.6]
Mind to body approaches, n (%) [95% CI]	320 (14.5%) [13.1 to 16.0]	242 (16.4%) [15.5 to 17.2]
**Life events / history, n (%) [95% CI]**		
History of medical conditions	1,700 (77.1%) [75.3 to 78.8]	1,161 (78.6%) [74.6 to 82.6]
Addiction (such as tobacco)	886 (40.2%) [38.1 to 42.2]	625 (42.3%) [40.2 to 44.5]
History of psychiatric disorders (such as anxiety and depression)	881 (39.9%) [37.9 to 42.0]	630 (42.7%) [40.5 to 44.8]

© 2026 Kerckhove N et al. *JAMA Network Open*.

Death of a relative	253 (11.5%) [10.2 to 12.9]	187 (12.7%) [12.0 to 13.3]
Accident	200 (9.1%) [7.9 to 10.3]	131 (8.9%) [8.4 to 9.3]
Divorce	190 (8.6%) [7.5 to 9.9]	139 (9.4%) [8.9 to 9.9]
Physical violence	181 (8.2%) [7.1 to 9.4]	132 (8.9%) [8.5 to 9.4]
Psychological violence	153 (6.9%) [5.9 to 8.1]	118 (8.0%) [7.6 to 8.4]
Burnout	131 (5.9%) [5.0 to 7.0]	97 (6.6%) [6.2 to 6.9]
Job loss	94 (4.3%) [3.5 to 5.2]	68 (4.6%) [4.4 to 4.8]
Sexual violence	93 (4.2%) [3.4 to 5.1]	68 (4.6%) [4.4 to 4.8]
Social isolation	89 (4.0%) [3.3 to 4.9]	62 (4.2%) [4.0 to 4.4]
**Questionnaire scores, mean (SD)**		
Injustice (/48)	27.2 (10.2)	27.2 (10.2)
Catastrophizing (/52)	26.5 (11.2)	26.3 (11.1)
Pain interference (/10)	4.0 (1.3)	4.0 (1.3)
Pain intensity (/10)	5.9 (1.9)	5.8 (1.9)
Kinesiophobia (/68)	42.0 (8.2)	41.9 (8.3)
Sleep quality (/100)	56.0 (17.4)	55.6 (17.2)
Cognition (/10)	4.9 (2.8)	4.9 (2.8)
Anxiety (/21)	10.0 (4.2)	10.0 (4.1)
Depression (/21)	8.1 (4.1)	8.0 (4.1)
Alexithymia (/100)	62.2 (10.6)	62.1 (10.7)
Social vulnerability (/100)	31.3 (20.4)	30.8 (20.6)
**Health related quality of life (EQ-5D-3L), mean (SD)**		
Daily activity (/3)	1.8 (0.5)	1.8 (0.5)
Anxiety (/3)	2.0 (0.7)	2.0 (0.7)
Autonomy (/3)	1.2 (0.4)	1.2 (0.4)
Pain (/3)	2.4 (0.5)	2.4 (0.5)
Mobility (/3)	1.5 (0.5)	1.5 (0.5)
Health quality (/100)	48.6 (20.1)	48.6 (20.0)

** Amitriptyline, Clomipramine, Duloxetine, Venlafaxine, Milnacipran, Imipramine*
*** Gabapentin, Pregabalin, Topiramate, Carbamazepine*
**** TENS, rTMS, spinal cord stimulation, transcutaneous vagus nerve stimulation (auricular, cervical) and cortical stimulation*

© 2026 Kerckhove N et al. *JAMA Network Open*.

eTable 2: Comparison of adherent and non-adherent patients. Baseline characteristics of patients who remained active in weekly monitoring versus those who discontinued reporting during follow-up.

Variables	Adherent (n = 602)			Non to Adherent* (n = 875)		
	n	mean / %	SD / [95%CI]	n	mean / %	SD / [95%CI]
**Sex and Body Mass Index**						
Female, n (%) [95% CI]	482	80.1%	[76.6 to 83.2]	731	83.5%	[80.9 to 85.9]
Male, n (%) [95% CI]	120	19.8%	[16.7 to 23.2]	144	16.3%	[14 to 19]
BMI, mean (SD)	602	27.5	6.8	875	27.3	7.1
BMI > 25, n (%) [95% CI]	342	56.8%	[52.8 to 60.8]	480	54.9%	[51.6 to 58.1]
**Age class, n (%) [95% CI]**						
18 to 24 years	13	2.2%	[1.2 to 3.7]	52	5.9%	[4.5 to 7.7]
25 to 34 years	54	9.0%	[6.8 to 11.5]	126	14.4%	[12.1 to 16.9]
35 to 49 years	218	36.2%	[32.4 to 40.2]	359	41.0%	[37.7 to 44.4]
50 to 64 years	266	44.2%	[40.2 to 48.3]	291	33.3%	[30.1 to 36.5]
≥65 years	51	8.5%	[6.4 to 11]	47	5.4%	[4 to 7.1]
**Family situation**						
In a relationship, n (%) [95% CI]	419	69.6%	[65.8 to 73.3]	595	68.0%	[64.8 to 71.1]
Single, n (%) [95% CI]	183	30.4%	[26.7 to 34.2]	280	32.0%	[28.9 to 35.2]
Number of children, mean (SD)	602	1.3	1.2	875	1.2	1.2
**Level of education, n (%) [95% CI]**						
Below high school diploma	168	27.9%	[24.4 to 31.7]	242	27.7%	[24.7 to 30.7]
High school diploma to Bachelor's degree	331	55.0%	[50.9 to 59]	449	51.3%	[47.9 to 54.7]
Above Bachelor's degree	103	17.1%	[14.2 to 20.4]	184	21.0%	[18.4 to 23.9]
**Employment status, n (%) [95% CI]**						
Employed	334	55.5%	[51.4 to 59.5]	535	61.1%	[57.8 to 64.4]
Unemployed	268	44.5%	[40.5 to 48.6]	340	38.9%	[35.6 to 42.2]
Disability allowance	62	10.3%	[8 to 13]	76	8.7%	[6.9 to 10.8]
Work to related disability	17	2.8%	[1.7 to 4.5]	14	1.6%	[0.9 to 2.7]
Non to work to related disability	156	25.9%	[22.5 to 29.6]	177	20.2%	[17.6 to 23]
Adapted work position	37	6.1%	[4.4 to 8.4]	39	4.5%	[3.2 to 6]
Recognition of disabled worker status	115	19.1%	[16 to 22.5]	111	12.7%	[10.6 to 15.1]
Sick leave related to pain	120	19.9%	[16.8 to 23.4]	202	23.1%	[20.3 to 26]
Sick leave unrelated to pain	17	2.8%	[1.7 to 4.5]	27	3.1%	[2 to 4.5]
**Tobacco and alcohol consumption, n (%) [95% CI]**						
Former smoker	359	59.7%	[55.7 to 63.7]	525	60.4%	[57.1 to 63.7]
Current smoker	117	19.5%	[16.4 to 22.9]	243	28.0%	[25 to 31.1]
Never smoked	125	20.8%	[17.6 to 24.3]	101	11.6%	[9.6 to 13.9]
No alcohol or less than once per month	235	39.1%	[35.2 to 43.1]	346	39.8%	[36.5 to 43.2]
Alcohol about once per month	154	25.6%	[22.2 to 29.3]	218	25.1%	[22.2 to 28.1]
Alcohol 2 to 4 times per month	151	25.1%	[21.7 to 28.8]	206	23.7%	[20.9 to 26.7]
Alcohol 2 to 3 times per week	44	7.3%	[5.4 to 9.7]	69	7.9%	[6.2 to 9.9]
Alcohol at least 4 times per week	17	2.8%	[1.7 to 4.5]	30	3.5%	[2.3 to 4.9]
**Chronic pain duration, n (%) [95% CI]**						
3 to 5 months	3	0.5%	[0.1 to 1.5]	8	0.9%	[0.4 to 1.8]
6 to 11 months	26	4.3%	[2.8 to 6.3]	47	5.4%	[4 to 7.1]
1 to 2 years	61	10.1%	[7.9 to 12.8]	121	13.9%	[11.7 to 16.4]

© 2026 Kerckhove N et al. *JAMA Network Open*.

3 to 4 years	100	16.6%	[13.7 to 19.9]	146	16.8%	[14.4 to 19.5]
5 to 10 years	171	28.5%	[24.9 to 32.2]	243	28.0%	[25 to 31.1]
More than 10 years	240	39.9%	[36 to 44]	304	35.0%	[31.8 to 38.3]
**Chronic pain diagnosis**						
Number of chronic pain diagnoses, mean (SD)	602	1.3	0.6	875	1.3	0.6
Nociplastic, n (%) [95% CI]	227	37.7%	[33.8 to 41.7]	321	36.7%	[33.5 to 40]
Musculoskeletal, n (%) [95% CI]	136	22.6%	[19.3 to 26.1]	177	20.2%	[17.6 to 23]
Peripheral neuropathic, n (%) [95% CI]	98	16.3%	[13.4 to 19.5]	106	12.1%	[10 to 14.5]
Post to surgical or post to traumatic, n (%) [95% CI]	93	15.4%	[12.7 to 18.6]	92	10.5%	[8.6 to 12.7]
Other, n (%) [95% CI]	84	14.0%	[11.3 to 17]	146	16.7%	[14.3 to 19.3]
Headache, n (%) [95% CI]	73	12.1%	[9.6 to 15]	104	11.9%	[9.8 to 14.2]
Visceral, n (%) [95% CI]	39	6.5%	[4.6 to 8.8]	101	11.5%	[9.5 to 13.8]
Central neuropathic, n (%) [95% CI]	8	1.3%	[0.6 to 2.6]	15	1.7%	[1 to 2.8]
Post to cancer treatment, n (%) [95% CI]	23	3.8%	[2.4 to 5.7]	34	3.9%	[2.7 to 5.4]
**Chronic pain pharmacological treatments**						
Number of analgesic treatments, mean (SD)	602	2.4	1.6	875	2.2	1.6
*Antidepressants recommended for the treatment of chronic pain**, n (%) [95% CI]*	250	41.5%	[37.6 to 45.6]	316	36.1%	[32.9 to 39.4]
Paracetamol, n (%) [95% CI]	246	40.9%	[36.9 to 44.9]	318	36.3%	[33.1 to 39.6]
Other, n (%) [95% CI]	196	32.6%	[28.8 to 36.5]	238	27.2%	[24.3 to 30.3]
*Anticonvulsants recommended for the treatment of chronic pain***, n (%) [95% CI]*	139	23.1%	[19.8 to 26.7]	182	20.8%	[18.2 to 23.6]
Opioids, n (%) [95% CI]	109	18.1%	[15.1 to 21.4]	167	19.1%	[16.5 to 21.8]
Anxiolytics/hypnotics, n (%) [95% CI]	80	13.3%	[10.7 to 16.3]	70	8.0%	[6.3 to 10]
NSAIDs, n (%) [95% CI]	74	12.3%	[9.8 to 15.2]	128	14.6%	[12.4 to 17.1]
Anesthetics, n (%) [95% CI]	64	10.6%	[8.3 to 13.4]	93	10.6%	[8.7 to 12.9]
Others antidepressants, n (%) [95% CI]	63	10.5%	[8.1 to 13.2]	79	9.0%	[7.2 to 11.1]
Capsaicin, n (%) [95% CI]	63	10.5%	[8.1 to 13.2]	88	10.1%	[8.1 to 12.2]
Triptans (anti to migraine), n (%) [95% CI]	46	7.6%	[5.6 to 10.1]	72	8.2%	[6.5 to 10.3]
Nefopam, n (%) [95% CI]	45	7.5%	[5.5 to 9.9]	58	6.6%	[5.1 to 8.5]
Ketamine, n (%) [95% CI]	27	4.5%	[3 to 6.5]	19	2.2%	[1.3 to 3.4]
Others Anticonvulsants, n (%) [95% CI]	20	3.3%	[2 to 5.1]	15	1.7%	[1 to 2.8]
Botulinum toxin, n (%) [95% CI]	16	2.7%	[1.5 to 4.3]	19	2.2%	[1.3 to 3.4]
Cannabinoids, n (%) [95% CI]	14	2.3%	[1.3 to 3.9]	16	1.8%	[1 to 3]
Others anti to inflammatory drugs, n (%) [95% CI]	11	1.8%	[0.9 to 3.2]	12	1.4%	[0.7 to 2.4]
Antirheumatic drugs, n (%) [95% CI]	1	0.2%	[0 to 0.9]	0	0.0%	[0 to 0.4]
**Chronic pain non to pharmacological treatments**						
Number of analgesic technics, mean (SD)	602	1.6	1.0	875	1.5	1.0
Physical and manual techniques, n (%) [95% CI]	284	47.2%	[43.1 to 51.3]	391	44.7%	[41.4 to 48.1]
*Neuromodulation****, n (%) [95% CI]*	280	46.5%	[42.5 to 50.6]	392	44.8%	[41.5 to 48.2]
Psychotherapeutic approaches, n (%) [95% CI]	115	19.1%	[16 to 22.5]	160	18.3%	[15.8 to 21]
Mind to body approaches, n (%) [95% CI]	104	17.3%	[14.3 to 20.5]	138	15.8%	[13.4 to 18.4]
Other, n (%) [95% CI]	157	26.1%	[22.6 to 29.8]	211	24.1%	[21.3 to 27.1]
**Life events / history, n (%) [95% CI]**						
History of medical conditions	498	82.7%	[79.5 to 85.7]	663	75.8%	[72.8 to 78.6]
Addiction (such as tobacco)	257	42.7%	[38.7 to 46.8]	368	42.1%	[38.8 to 45.4]
History of psychiatric disorders (such as anxiety and depression)	283	47.0%	[43 to 51.1]	347	39.7%	[36.4 to 43]
Death of a relative	77	12.8%	[10.2 to 15.7]	110	12.6%	[10.4 to 15]

© 2026 Kerckhove N et al. *JAMA Network Open*.

Divorce	59	9.8%	[7.5 to 12.5]	80	9.1%	[7.3 to 11.2]
Accident	56	9.3%	[7.1 to 11.9]	75	8.6%	[6.8 to 10.6]
Burnout	44	7.3%	[5.4 to 9.7]	53	6.1%	[4.6 to 7.8]
Physical violence	42	7.0%	[5.1 to 9.3]	90	10.3%	[8.4 to 12.5]
Psychological violence	42	7.0%	[5.1 to 9.3]	76	8.7%	[6.9 to 10.8]
Job loss	29	4.8%	[3.2 to 6.8]	39	4.5%	[3.2 to 6]
Social isolation	24	4.0%	[2.6 to 5.9]	38	4.3%	[3.1 to 5.9]
Sexual violence	22	3.7%	[2.3 to 5.5]	46	5.3%	[3.9 to 7]
**Questionnaire scores, mean (SD)**						
Injustice (/48)	602	26.6	10.3	875	27.5	10.0
Catastrophizing (/52)	602	25.8	11.2	875	26.6	11.1
Pain interference (/10)	602	40.1	13.5	875	40.4	12.9
Pain intensity (/10)	602	5.8	1.9	875	5.8	1.9
Kinesiophobia (/68)	602	42.1	8.3	875	41.8	8.4
Sleep quality (/100)	602	54.1	17.9	875	56.6	16.7
Cognition (/10)	602	4.9	2.8	875	4.9	2.8
Anxiety (/21)	602	9.6	4.1	875	10.3	4.0
Depression (/21)	602	8.0	4.1	875	8.1	4.1
Alexithymia (/100)	602	62.4	11.2	875	61.9	10.3
Social vulnerability (/100)	602	29.7	20.7	875	32.0	20.5
**Health related quality of life questionnaire (EQ-5D-3L), mean (SD)**						
Daily activity (/3)	602	1.8	0.5	875	1.8	0.5
Anxiety (/3)	602	2.0	0.7	875	2.0	0.7
Autonomy (/3)	602	1.2	0.4	875	1.2	0.4
Pain (/3)	602	2.4	0.5	875	2.4	0.5
Mobility (/3)	602	1.5	0.5	875	1.5	0.5
Health quality (/100)	602	49.2	20.7	875	48.1	19.4

** Non to adherence was defined as the absence of any recorded data during the 6th month of follow to up (between weeks 24 to 28)*
*** Amitriptyline, Clomipramine, Duloxetine, Venlafaxine, Milnacipran, Imipramine*
**** Gabapentin, Pregabalin, Topiramate, Carbamazepine*
***** TENS, rTMS, spinal cord stimulation, transcutaneous vagus nerve stimulation (auricular, cervical) and cortical stimulation*

© 2026 Kerckhove N et al. *JAMA Network Open*.

eTable 3: Statistical Performance and Selection Criteria for Multivariable Gaussian Mixture Models. This table summarizes the fit indices and classification accuracy for multivariable Gaussian Mixture Models ranging from one to four classes. G: number of latent classes; loglik: log-likelihood; npm: number of estimated parameters; BIC: Bayesian Information Criterion (lower values indicate a better balance between model fit and parsimony); %class: estimated proportion of participants in each latent cluster. Entropy measures the overall quality of class separation (ranging from 0 to 1, with values closer to 1 indicating better separation). The 3-class model was selected as the optimal solution based on the elbow in the BIC curve and the clinical interpretability of the identified trajectories, whereas the 4-class model showed evidence of overfitting with the creation of a micro-cluster (< 1% of the sample).

	G	loglik	nfp	BIC	n (%) class1 [95% CI]	n (%) class2 [95% CI]	n (%) class3 [95% CI]	n (%) class4 [95% CI]	Entropy
**model 1**	1	-36804	10	73682	1477 (100.0)	x	x	x	
**model 2**	2	-36643	13	73381	1127 (76.3) [74.1% to 78.5%]	350 (23.7) [21.5% to 25.9%]	x	x	0.35
**model 3**	3	-36605	16	73327	1266 (85.7) [83.9% to 87.5%]	62 (4.1) [3.2% to 5.2%]	150 (10.2) [8.6% to 11.7%]	x	0.52
**model 4**	4	-36594	19	73327	1211 (82.0) [80.0% to 84.0%]	8 (0.6) [0.2% to 1.0%]	44 (3.0) [2.1% to 3.8%]	214 (14.5) [12.7% to 16.3%]	0.56

G, number of components; loglik, log-likelihood; nfp, Number of free parameters; BIC, Bayesian Information Criterion

© 2026 Kerckhove N et al. *JAMA Network Open*.

eTable 4: Performance metrics of predictive models for trajectory class membership. Predictive performance of machine learning models trained on baseline sociodemographic and clinical variables to classify patients into trajectory groups. Metrics include accuracy, balanced accuracy, Cohen's kappa, F1-score, sensitivity and specificity for minority classes.

Model	Accuracy ±SD	Kappa ±SD	Balanced Accuracy ±SD	F1 score ±SD	Sensitivity ±SD	Specificity ±SD
**Multinomial**	0.53 ± 0.04	0.05 ± 0.04	0.55 ± 0.05	0.37 ± 0.03	0.48 ± 0.07	0.71 ± 0.03
**KNN (SMOTE)**	0.39 ± 0.03	0.01 ± 0.03	0.51 ± 0.04	0.28 ± 0.06	0.39 ± 0.06	0.69 ± 0.04
**SVM (SMOTE)**	0.85 ± 0.01	0.05 ± 0.05	0.51 ± 0.03	0.52 ± 0.19	0.35 ± 0.01	0.68 ± 0.01
**Random Forest**	0.86 ± 0.00	0.00 ± 0.01	0.50 ± 0.00	0.92 ± 0.00	0.33 ± 0.00	0.67 ± 0.00
**RF balanced**	0.86 ± 0.00	0.00 ± 0.01	0.50 ± 0.00	0.92 ± 0.00	0.33 ± 0.00	0.67 ± 0.00
**XGBoost**	0.85 ± 0.01	0.04 ± 0.08	0.51 ± 0.03	0.91 ± 0.20	0.33 ± 0.03	0.66 ± 0.02
**CatBoost**	0.79 ± 0.02	0.17 ± 0.05	0.58 ± 0.02	0.44 ± 0.11	0.44 ± 0.05	0.72 ± 0.02

KNN, k Nearest Neighbors; SVM, Support-Vector Machine; SMOTE, Synthetic Minority Oversampling Technique; eXtreme Gradient Boosting, XGBoost ; CatBoost, Categorical Boosting; SD, standard deviation

© 2026 Kerckhove N et al. *JAMA Network Open*.
