# Supplementary material for: Digital Monitoring and Chronic Pain Trajectories in Patients Receiving Tertiary Pain Care
Source: JAMA Netw Open. 2026 Aug 4;9(8):e2627217. doi: 10.1001/jamanetworkopen.2026.27217 (PMC13439428; doi:10.1001/jamanetworkopen.2026.27217)
Supplement: Supplementary file 2 [file jamanetwopen-e2627217-s002.pdf]

Data Sharing Statement

Kerckhove. Digital Monitoring and Chronic Pain Trajectories in Patients Receiving Tertiary Pain Care. *JAMA Netw Open*. Published August 04, 2026.
doi:10.1001/jamanetworkopen.2026.27217

Data
Data available: Yes
Data types: Other (please specify)
Additional Information: R code for analysis and cohort data
How to access data: nkerckhove@chu-clermontferrand.fr
When available: With publication

Supporting Documents
Document types: Statistical/analytic code
How to access documents: nkerckhove@chu-clermontferrand.fr
When available: With publication

Additional Information
Who can access the data: researchers whose proposed use of the data has been approved
Types of analyses: for any purpose
Mechanisms of data availability: with a signed data access agreement
